# A six-mRNA signature model for the prognosis of head and neck squamous cell carcinoma

**DOI:** 10.18632/oncotarget.21786

**Published:** 2017-10-10

**Authors:** Wenna Guo, Xijia Chen, Liucun Zhu, Qiang Wang

**Affiliations:** ^1^ State Key Laboratory of Pharmaceutical Biotechnology, School of Life Sciences, Nanjing University, Nanjing, China; ^2^ School of Life Sciences, Shanghai University, Shanghai, China

**Keywords:** biomarker, HNSCC, prognosis, risk-stratification, RNA-seq

## Abstract

Head and neck squamous cell carcinoma (HNSCC), one of the most common cancers with high morbidity and mortality rates worldwide, has a poor prognosis. The transcriptome sequencing data of 500 patients with HNSCC in the TCGA dataset were assessed to find biomarkers associated with HNSCC prognosis so as to improve the prognosis of patients with HNSCC. The patients were divided into the training and testing sets. A model of six mRNAs (FRMD5, PCMT1, PDGFA, TMC8, YIPF4, ZNF324B) that could predict patient prognosis was identified in the training set using the Cox regression analysis. According to this model, the patients were divided into high-risk and low-risk groups. The Kaplan-Meier analysis showed that the high-risk group showed significantly shorter overall survival time compared with the low-risk group in both training and testing sets. The receiver operating characteristic analysis further confirmed high sensitivity and specificity for the model, which was more accurate compared with some known biomarkers in predicting HNSCC prognosis. Moreover, the model was applicable to patients of different ages, genders, clinical stages, tumor locations, smoking history, and human papillomavirus (HPV) status, as well as to microarray dataset. This model could be used as a novel biomarker for the prognosis of HNSCC and a significant tool for guiding the clinical treatment of HNSCC. The risk score acquired from the model might contribute to improving outcome prediction and management for patients with HNSCC, indicating its clinical significance.

## INTRODUCTION

Head and neck cancer is a malignancy that originates in the oral cavity, tongue, lip, gum, oropharynx, nasopharynx, and hypopharynx [1]. More than 90% of head and neck cancers are head and neck squamous cell carcinoma (HNSCC) [2]. HNSCC ranks sixth in incidence and eighth in mortality among all cancers. Global cancer estimates indicate that as many as 0.6 million new cases of HNSCC were reported in 2012 worldwide, accounting for about 4.3% of all cancer cases [3]. The incidence of this highly malignant cancer has shown an increasing trend in recent years. Although HNSCC diagnosis and treatment have greatly improved with the rapid development of medical technology, patient prognosis remains low. Indeed, the 5-year survival rate of patients with HNSCC is less than 50% [4]. Early-stage HNSCC cases have been treated mainly by surgery and radiation therapy over the past few decades, while the therapeutic strategy for advanced HNSCC is a combination of surgery, radiotherapy, and chemotherapy. It has been clinically demonstrated that a combination of radiotherapy and chemotherapy has certain effects on cancers, but the improvement in treatment outcomes is limited [5, 6]. For example, the survival rate is only slightly increased, and cancer recurrence and treatment failure still occur in a significant proportion of patients [7]. Therefore, it is imperative to identify novel and reliable markers that can predict patient prognosis to distinguish patients with different risks. Then, the patient prognosis can be improved by selecting appropriate treatment.

Protein-encoding mRNA is one of the most common molecular markers. Multiple studies demonstrated its involvement in a variety of cancers as well as association with patient prognosis. For instance, it was found that zinc finger E-box binding homeobox 2 was highly expressed in ovarian cancer tissues, with this high expression level significantly associated with a worse prognosis [8]. Forkhead box Q1 was upregulated in pancreatic cancer, and its expression was negatively correlated with patient survival [9]. As for HNSCC, studies also showed that gene expression might provide valuable information for patient prognosis. Zheng et al. found that forkhead box F2 was downregulated in esophageal squamous cell carcinoma, and the reduced levels were related to poor prognosis [10]. Clauditz et al. demonstrated that U3 small nucleolar ribonucleoprotein is overexpressed in various cancers, with the level significantly associated with survival in patients with HNSCC [11]. However, previous studies mostly focused on the relationship between a single mRNA and cancer, and lacked uniform standards due to the limitation of observer variability. In addition, the expression of a single gene might be unstable, resulting in a high rate of false positives. For instance, Chiarelli et al. found that carbohydrate antigen 19-9 (CA19-9) and carcinoembryonic antigen (CEA) could not be used as independent prognostic factors in gastric cancer. Indeed, CA19-9 and CA125 (also known as mucin 16) had the highest sensitivity and specificity, respectively, and a combination of CA19-9 and CEA was shown to be the best for predicting patient prognosis. However, detailed studies assessing the expression levels of multiple genes as prognostic biomarkers for HNSCC are scarce.

In this study, the Cox regression analysis was used to assess HNSCC RNA-seq data in TCGA. Subsequently, the association of patient survival time with the expression levels of multiple genes was analyzed, and a proportional hazards model was established to predict patient survival. Furthermore, the reliability of the model and its application value were assessed in patients of different ages, genders, clinical stages, smoking history and human papillomavirus (HPV) status using Kaplan-Meier and receiver operating characteristic (ROC) analyses.

## RESULTS

### Clinical characteristics of the patients

After selection, a total of 500 patients were included in this study, with an average age of 60 years (ranging from 19 to 90 years). The average overall survival (OS) time was 743 days. Most patients were male (*n* = 369 out of 500, 73.8%), and 75.2% of them were smokers. Different patients had a different smoking history: 173 were current smokers, 71 were current reformed smokers for ≤15 years, 132 were current reformed smokers for >15 years, and 108 were nonsmokers. The number of packs smoked per year ranged from 0.17 to 300, and the median was 40 packs. According to the American Joint Committee on Cancer staging system, 20, 97, 100, and 270 patients were in stages I, II, III, and IV, respectively. Meanwhile, the study specimens originated from 13 different sites in patients, including tongue, oral cavity, pharynx, tonsil, and larynx; 125, 114, 71, and 43 originated from tongue, larynx, oral cavity, tonsil, respectively. In addition, 37 patients were HPV positive, and 72 patients were HPV negative. HPV-negative patients showed significantly higher mortality rates (chi-square test, *P* < 0.05). The detailed clinical information of patients is summarized in Table 1 and Supplementary Table 1.

**Table 1:** The main demographic, clinical and pathological characteristics of the 500 HNSCC patients from TCGA

Characteristic		Num	Percentage	Dead num	χ^2a^	*P*-value^b^
Age	≤60	250	50%	86	4.077	0.043
	>60	250	50%	108		
Gender	Female	131	26.20%	62	8.625	0.003
	Male	369	73.80%	132		
Stage	Stage I	20	4.11%	7	0.668	0.881
	Stage II	97	19.92%	38		
	Stage III	100	20.53%	43		
	Stage IV	270	55.44%	106		
Smoked time	≤15 years	132	34.29%	54	5.068	0.167
	>15 years	71	18.44%	29		
	current smoker	173	18.96%	69		
	Non-smoker	109	28.31%	33		
Smoked packs	≤40 packs	163	32.60%	57	0.798	0.777
	>40 packs	123	24.60%	45		
HPV status	Positive	37	7.40%	3	4.483	0.034
	Negative	72	14.40%	18		

a. χ^2^ refers to the Pearson Chi-Square value.

b. *P*-value refers to the significant level in Chi-Square test.

### Identification of survival-related genes using the training dataset

A univariate Cox regression analysis was used to explore the relationship between gene expression level and patient survival in the training set so as to identify survival-related genes. A total of 252 genes were identified to be significantly associated with the OS of patients (*P* < 0.001), of which 91 showed differential expression between patients with longer OS and those with shorter OS time (*P* < 0.05) (Supplementary Table 2). Of these 91 genes, 39 genes were selected by screening the genes that were protein coding and expressed in at least half of the HNSCC tissues. Then, 2-6 genes from 39 genes were selected as covariates to perform a multivariate Cox stepwise regression analysis. A model consisting of six mRNAs was determined after comparisons. The model could be used for survival prediction. The risk scoring formula of these six mRNAs was as follows: Risk score = 0.072 × expression value of FERM domain containing 5 (FRMD5) + 0.512 × expression value of (protein-L-isoaspartate (D-aspartate) O-methyltransferase (PCMT1) + 0.201 × expression value of platelet-derived growth factor subunit A (PDGFA) – 0.158 × expression value of transmembrane channel like 8 (TMC8) + 0.615 × expression value of Yip1 domain family member 4 (YIPF4) – 0.145 × expression value of zinc finger protein 324B (ZNF324B). Obviously, high expression levels of FRMD5, PCMT1, PDGFA, and YIPF4, but low expression levels of TMC8 and ZNF324B, were related to a higher risk. This was consistent with the aforementioned results of differential expression. The chromosomal positions of these six mRNAs are shown in Table 2.

**Table 2:** Six mRNAs significantly related to the overall survival of patients in the training set (*n* = 259)

Gene symbol	Location	Length	*P* value^a^	HR^a^ (95% CI)	Coefficient^b^	Differential expression^c^
FRMD5|84978	15q15.3	2560	3.11E-04	1.228 (1.10-1.37)	0.072	0.0088
PCMT1|5110	6q25.1	1888	6.92E-05	2.308 (1.53-3.49)	0.512	0.0099
PDGFA|5154	7p22	2804	1.80E-04	1.512 (1.22-1.88)	0.201	0.001
TMC8|147138	17q25.3	4417	5.77E-04	0.738 (0.62-0.88)	-0.158	0.0064
YIPF4|84272	2p22.3	1963	4.66E-04	2.371 (1.46-3.84)	0.615	0.019
ZNF324B|388569	19q13.43	3812	6.63E-04	0.548 (0.39-0.77)	-0.145	0.021

a. The log-rank test in the univariate Cox regression analysis.

b. The coefficient value of the gene in the prognostic model of six mRNAs derived from the multivariate Cox regression analysis.

c. The value of differential expression significant level between long-term (>36 months) and short-term (<12 months) survival patients.

### Relationship between the six-mRNA signature and patient survival

**Figure 1:**
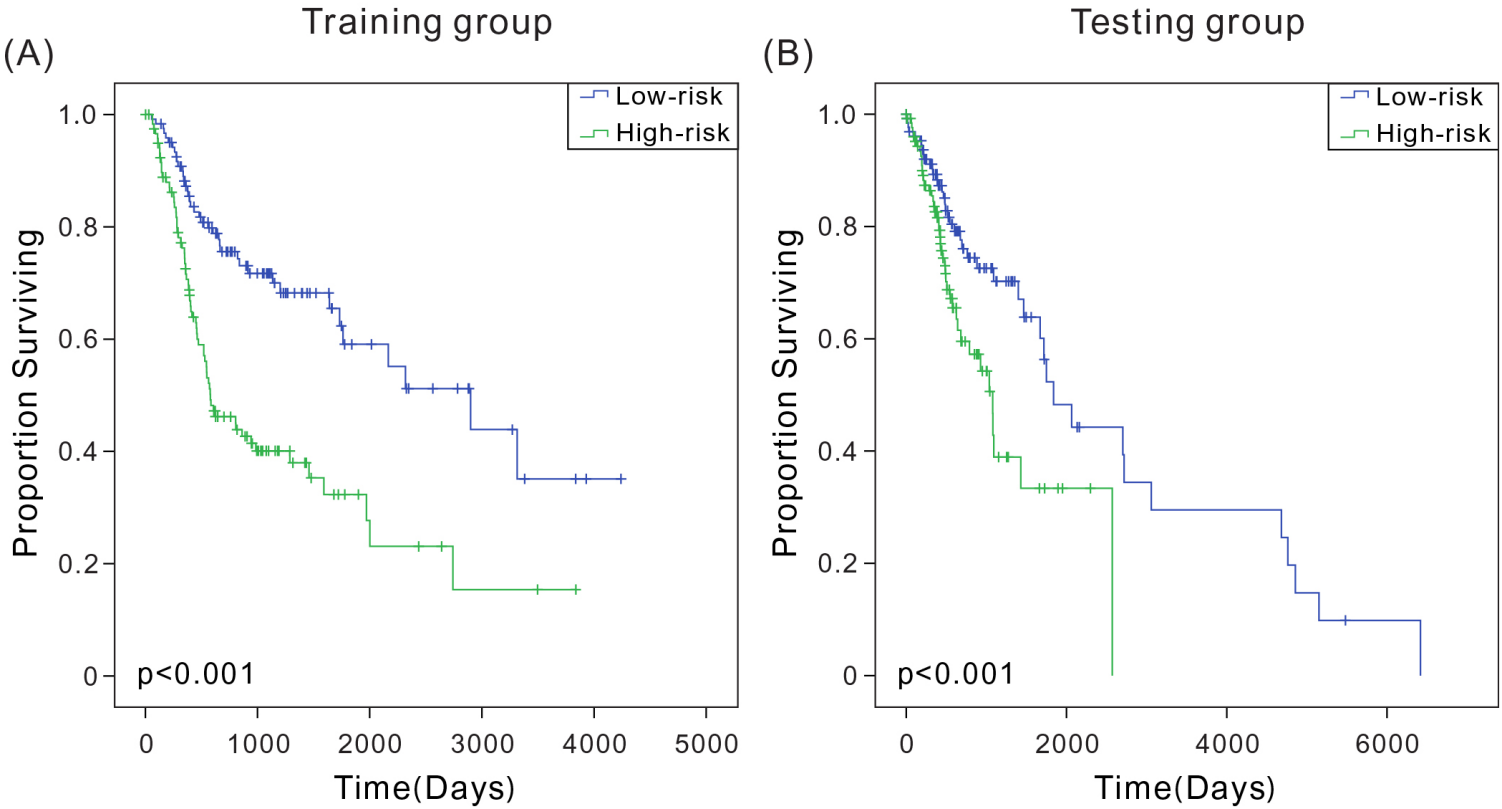
Kaplan-Meier analysis with two-sided log-rank test estimates of the survival of TCGA patients using the six-mRNA signature **(A)** Kaplan-Meier curves for the training-set patients (*n* = 241); **(B)** Kaplan-Meier curves for the testing-set patients (*n* = 259).

Hazard ratios (HRs) from the Cox regression analysis showed that the six-mRNA signature was significantly associated with patient survival (*P* < 0.0001, HR: 2.718, 95% CI: 2.004-3.686). The Kaplan-Meier survival analysis was used to evaluate the relationship between this signature and patient OS so as to determine the potential predictive value of six-mRNA signature in the prognosis of patients with HNSCC. According to the six-mRNA risk scoring formula, risk scores were calculated for patients in the training set. As in previous reports [12, 13], the patients were ranked according to their risk scores and divided into high-risk (*n* = 120) and low-risk (*n* = 121) groups using the median risk score as the cutoff point. The low-risk and high-risk groups corresponded to patients with relatively low score and high score of six-mRNA signature, respectively. The combined Kaplan-Meier analysis and long-rank test revealed that the average OS of patients in the high-risk and low-risk groups was 716 and 1074 days, indicating a significant difference between the two groups (log-rank test, *P* < 0.001) (Figure 1A). Similar results were obtained for the testing set (Figure 1B). Therefore, this model could accurately predict patient survival. The predicted high-risk patients had relatively shorter OS, suggesting that the six-mRNA signature model had a great predictive value for HNSCC prognosis.

### Evaluation of the predictive performance of the six-mRNA signature model by ROC analysis

**Figure 2:**
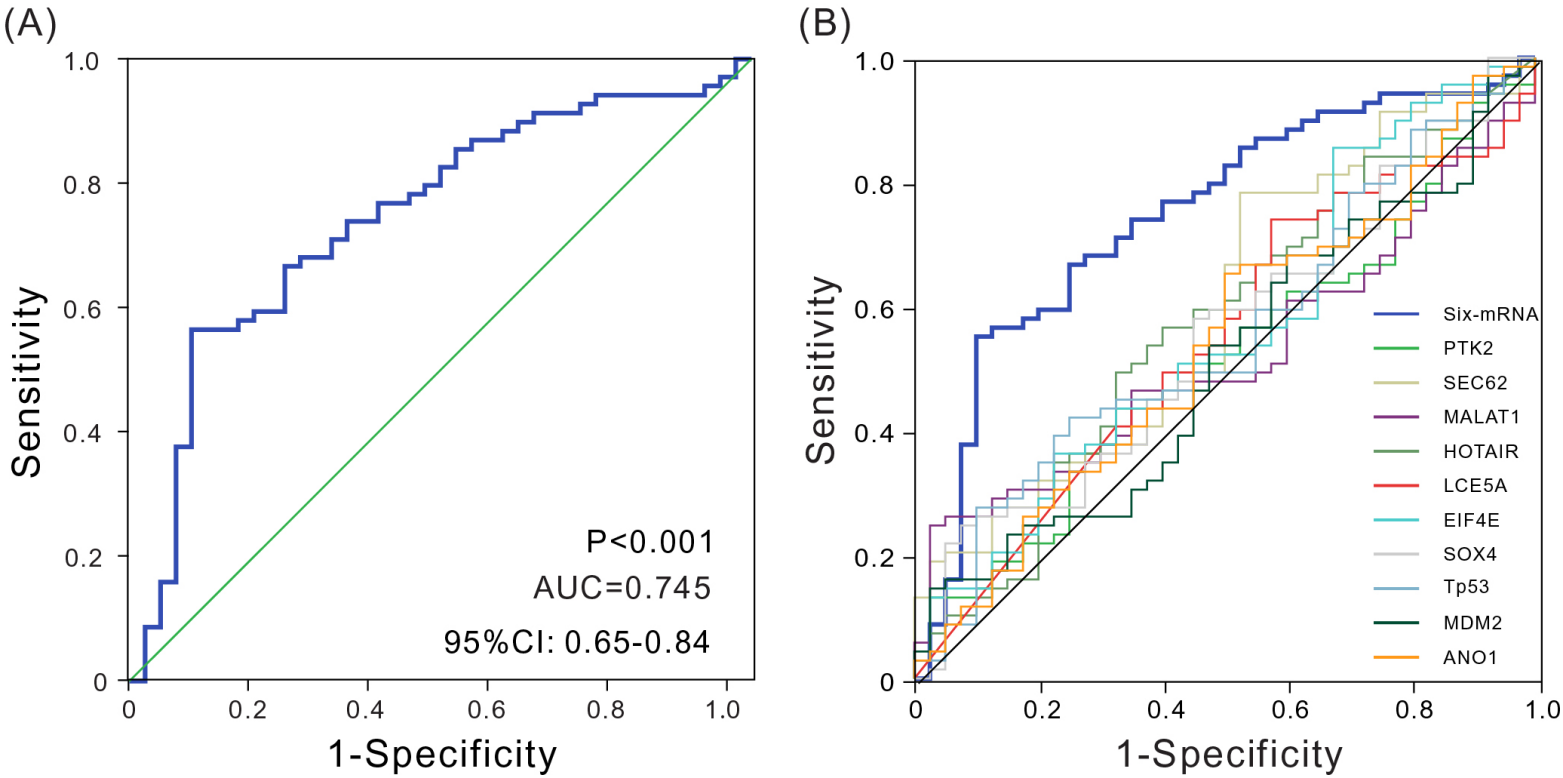
ROC analysis of sensitivity and specificity **(A)** ROC analysis of sensitivity and specificity using the six-mRNA signature in predicting the patient overall survival, AUC = 0.745 (*P* < 0.001). **(B)** ROC curves show the sensitivity and specificity of different biomarkers in predicting the patient overall survival.

An ROC curve analysis was employed to evaluate the sensitivity and specificity of the six-mRNA signature model in predicting survival in the testing set so as to further verify its accuracy of survival prediction. The area under the curve (AUC) was determined using 3 years as the cutoff point. As shown in Figure 2A, AUC was 0.745 (*P* < 0.001, 95% CI: 0.648-0.842), suggesting that the six-mRNA signature model had relatively high sensitivity and specificity, and therefore could be regarded as a prognostic prediction factor to estimate survival risk of patients. Thus, it provided a novel reference strategy for preoperative differential diagnosis and postoperative differential treatment in patients, further indicating the clinical value of this signature.

### Comparison of the novel signature model with other prognostic biomarkers in terms of predictive performance

Previous studies have identified a number of HNSCC prognostic biomarkers. For example, Reddy et al. found that anoctamin 1 was significantly associated with HNSCC patient survival. Singh et al. found that eukaryotic translation initiation factor 4E) was a prognostic marker for patients with HNSCC. An ROC analysis was used to compare the sensitivity and specificity of six-mRNA signature model with those of known HNSCC prognostic biomarkers so as to further assess the model for its performance in predicting patient survival. Interestingly, the six-mRNA signature model showed a significantly higher AUC value compared with the values obtained for the known biomarkers assessed. These results indicated the superiority of novel signature over the tested biomarkers for predicting OS in HNSCC (Figure 2B and Supplementary Table 3).

### Universality of the six-mRNA signature model in patients with different ages, genders, clinical stages, smoking history, and HPV status

**Figure 3:**
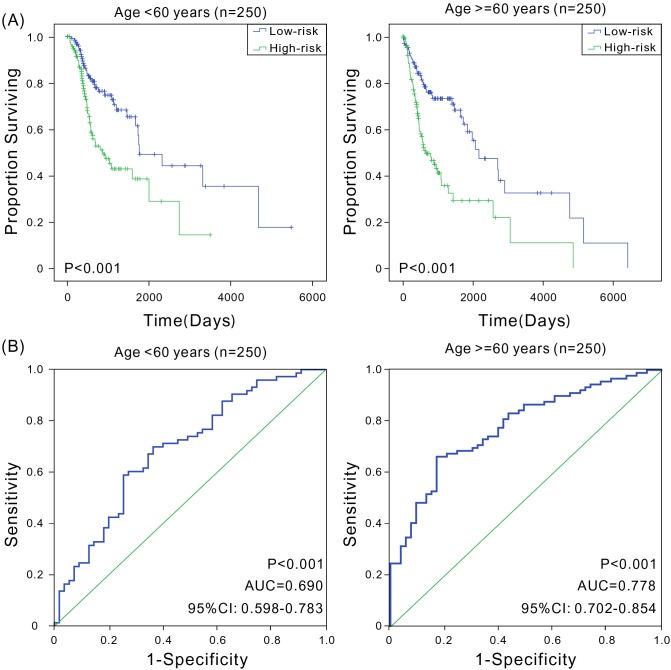
Kaplan-Meier and ROC analyses for the overall survival of patients in different age groups **(A)** Kaplan-Meier estimates of the overall survival of patients with different age. The survival differences between the two curves were determined by the two-sided log-rank test; **(B)** ROC curves show the sensitivity and specificity of the six-mRNA signature in predicting the patient overall survival.

**Figure 4:**
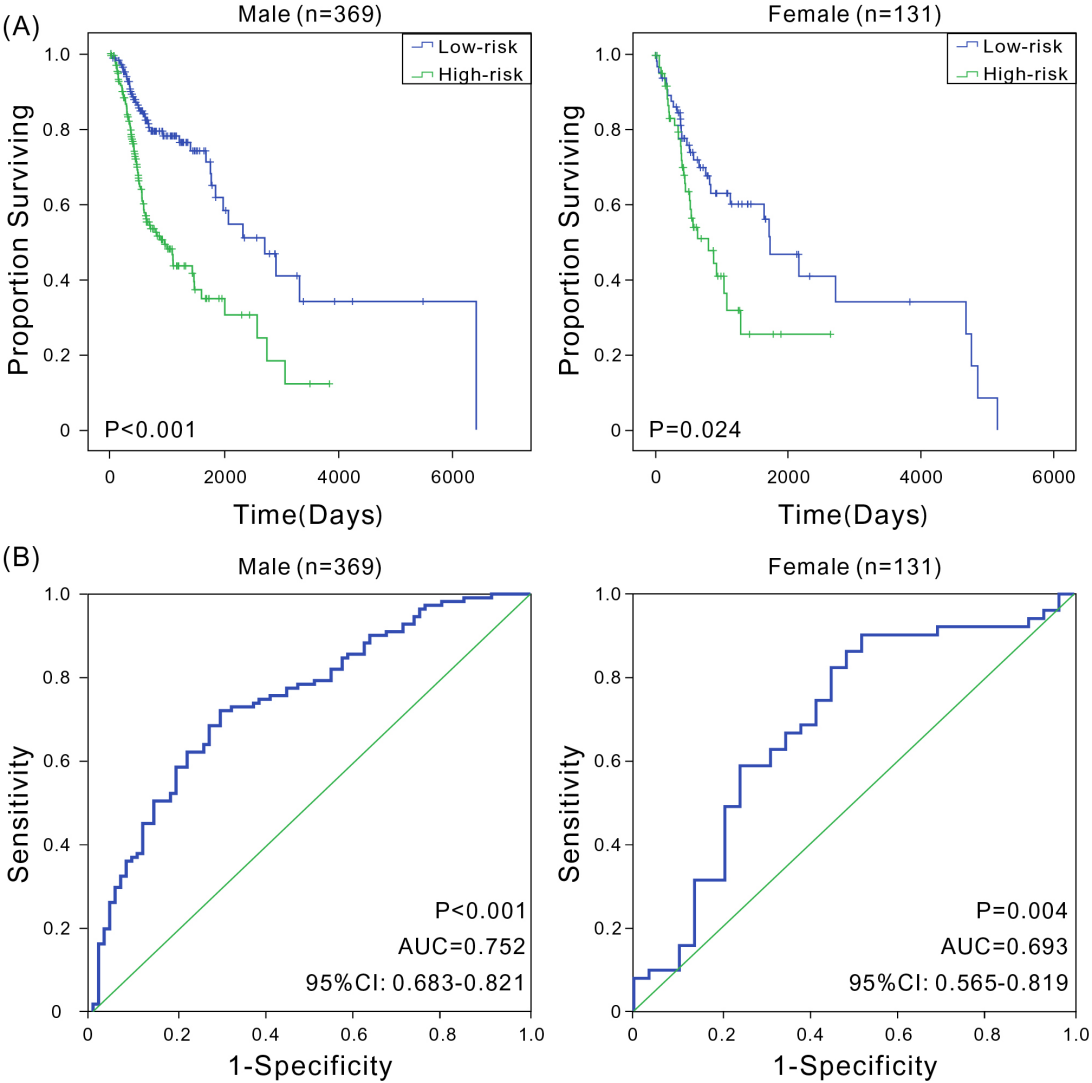
Kaplan-Meier and ROC analyses for the overall survival of patients in different gender groups **(A)** Kaplan-Meier estimates of the overall survival of patients with different gender; **(B)** ROC curves show the sensitivity and specificity of the six-mRNA signature in predicting the patient overall survival.

A variety of factors such as age [14], gender [3, 15], clinical stage [16], smoking history [17, 18], and HPV status [19] can also affect patient prognosis. This study analyzed the prognostic value of six-mRNA signature model in various groups. First, younger patients were found to have a better prognosis [14]. The patients were divided into low-age (age ≤60 years, *n* = 250) and high-age groups (age >60 years, *n* = 250) based on the median of patients’ ages. The Kaplan-Meier and ROC analyses revealed that patients in the high-risk group had significantly shorter OS compared with patients in the low-risk group in both low-age and high-age groups (*P* < 0.001), with good predictive performance (AUC was 0.690 and 0.778, respectively) (Figure 3). Second, male patients had higher mortality rates compared with females [3], and in this study, the OS of male patients (691 days) was significantly shorter than that of females (849 days). Therefore, male and female patients were assessed separately. In both groups, high-risk patients had significantly shorter OS (*P* < 0.001), with the AUC values of 0.752 and 0.693, respectively (Figure 4), indicating that the six-mRNA signature model was applicable in both gender groups. Next, the patients were grouped according to the clinical stage. Considering that stages I and II had small sample sizes, patients in both stages were combined into a single group. The Kaplan-Meier and ROC analyses found that the six-mRNA signature model also had universality in groups with different stages (Supplementary Figure 1). Studies have shown that smoking is also an important factor affecting patient prognosis. Indeed, smokers generally have TP53 loss of function and reduced copy number [20]. In this study, reformed smokers for >15 years showed significantly shorter OS compared with nonsmokers (*t* test, *P* < 0.01), and patients with more than 40 packs smoked per year and patients with not more than 40 packs smoked per year had significant different OS (*t* test, *P* < 0.001). The Kaplan-Meier and ROC analyses were performed to analyze the value of six-mRNA signature model in patients with a smoking history. Furthermore, regardless of the smoking history, patients in the high-risk group showed significantly shorter OS compared with patients in the low-risk group (*P* < 0.05), and the ROC analysis also indicated that the six-mRNA signature model had good sensitivity and specificity (AUC > 0.7) (Supplementary Figures 2-3). Since the signature might have different adaptability for various HNSCC sites [21, 22], the predictive performance of six-mRNA signature model was assessed in pharyngeal and tongue cancers, and high sensitivity and specificity were found for both; in tongue cancer, the AUC was as high as 0.806 (Supplementary Figure 4). In addition, HPV-positive patients were more likely than HPV-negative patients to have better survival [19]. The six-mRNA signature could distinguish high-risk patients from low-risk patients with high accuracy among both HPV-positive patients and HPV-negative patients (Supplementary Figure 5). Taken together, these results indicated that the six-mRNA signature model was suitable for patients with the HNSCC of different ages, genders, clinical stages, smoking history, HPV status, and tumor sites, further illustrating its important clinical value.

### Validation of the six-mRNA signature model for survival prediction using GEO dataset

**Figure 5:**
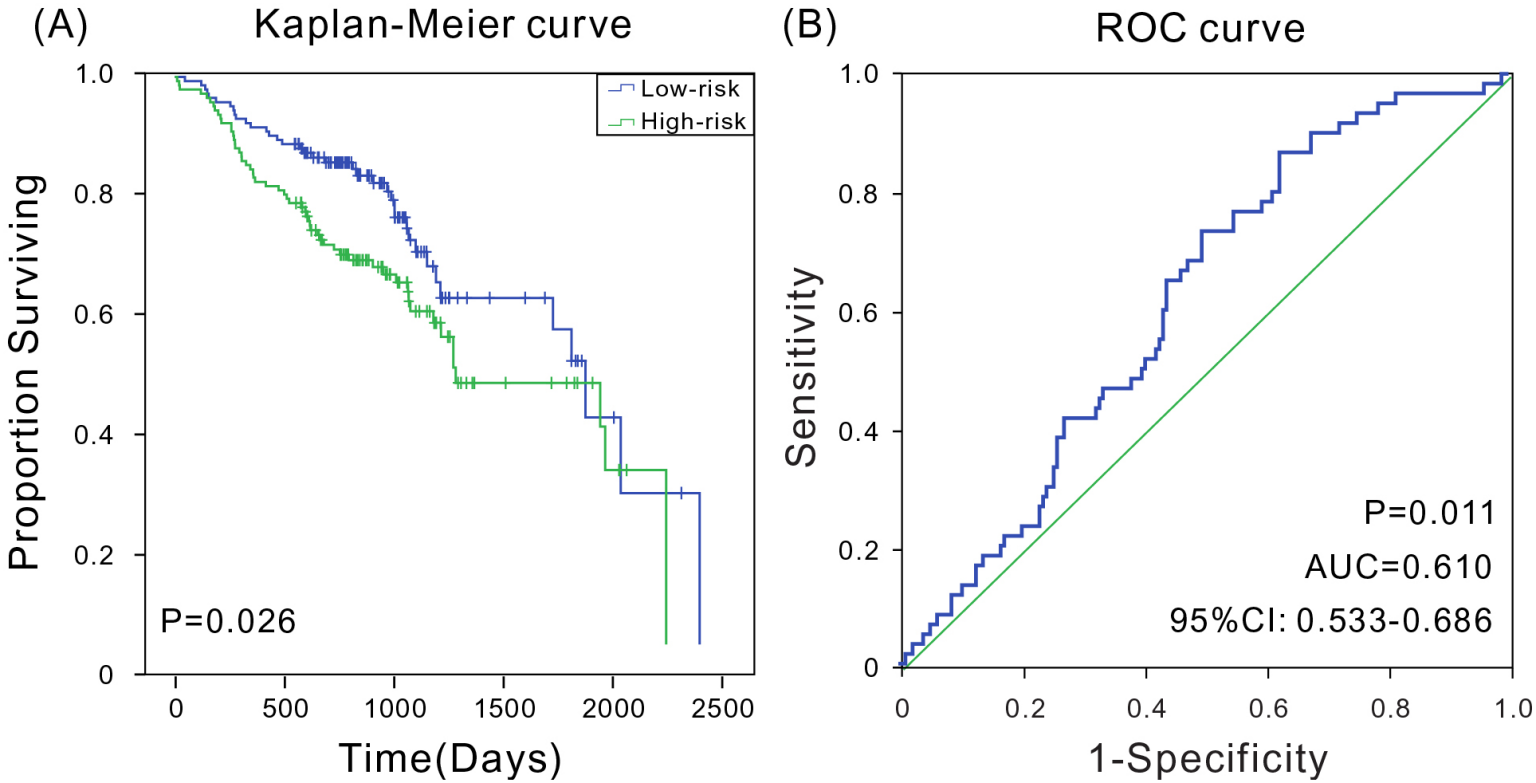
Kaplan-Meier and ROC analyses for the overall survival of patients from GSE65858 **(A)** Kaplan-Meier survival curves show a correlation between the expression of six-mRNA signature model and overall survival of patients; **(B)** ROC curves show the sensitivity and specificity of the six-mRNA signature model in predicting the patient overall survival, AUC = 0.610 (*P* < 0.05).

In addition, microarray expression data and corresponding clinical data of 270 patients with HNSCC from National Center for Biotechnology Information GEO dataset (GSE65858, Illumina HumanHT-12 V4.0 expression beadchip)[23] were downloaded to further verify whether the prognostic marker was applicable to other datasets. The Kaplan-Meier analyses indicated that the model could accurately distinguish high-risk patients from low-risk patients (*P* = 0.026) (Figure 5A). The ROC analysis indicated that the six-mRNA signature had high sensitivity and specificity (AUC = 0.610, 95% CI: 0.533-0.686) in this expression profile chip dataset (Figure 5B). Therefore, the results indicated that the six-mRNA signature model still performed well in prognosis prediction on microarray dataset.

## DISCUSSION

Recent studies demonstrated that mRNAs could be used as molecular markers for cancer development and prognosis, indicating their important clinical significance. De Melo et al. found that SH3 and multiple ankyrin repeat domains (SHANK)-associated RH domain interactor was overexpressed during breast cancer development, and its suppression could inhibit breast cancer [24]. Han et al. confirmed that MAGE family member A9 had significantly higher expression in laryngeal squamous cell carcinoma and could be used as an independent prognostic factor in patients with laryngeal squamous cell cancer [25]. Lou et al. used a Cox proportional hazards regression analysis to demonstrate that patients with liver cancer having the high expression of SRY-box 1 (SOX-1) had a better prognosis, and the SOX-1 status could be used as a prognostic factor in patients with liver cancer [26]. In this study, a univariate Cox regression analysis was used to identify mRNAs associated with the survival of patients with HNSCC. A six-mRNA signature model that could predict patient survival was identified by a multivariate Cox proportional hazards model analysis. It was confirmed that the model was also applicable in the microarray dataset. Compared with some known prognostic biomarkers, this model showed better performance on outcome prediction in patients with HNSCC, indicating a higher utility and important clinical value of the proposed six-mRNA signature.

Studies demonstrated that HNSCCs originating from distinct sites had differences at the molecular level. Consequently, a prognostic marker might not be universal. For instance, the expression of CD44 predicted survival in patients with pharyngeal and laryngeal tumors, but not in those with oral cancer [21, 22]. Therefore, it was clinically significant to develop prognostic markers with high universality. This study demonstrated that the six-mRNA signature model was applicable to patients of different ages, genders, clinical stages, and degrees of smoking. For different age and gender groups, the six-mRNA signature model could well distinguish between the high-risk and low-risk groups (*P* < 0.05). Thus, different treatments could be chosen, which was of great significance. The currently known cancer staging systems have many limitations for different clinical stages, and additional prognostic factors based on patient risk classification at the molecular level can help provide an optimized and personalized cancer treatment. The six-mRNA signature model could well distinguish patients with different survival risks, and even those with early disease stage could be segregated. Different treatments could be selected according to the varying risk levels, improving patient survival. These findings provided a potential guideline for choosing clinical treatment for patients with HNSCC in the early stage. The six-mRNA signature model was also efficient in different patient groups according to the smoking status. The AUC of six-mRNA signature model was 0.610 for 270 patients with HNSCC in the microarray dataset, exhibiting false positivity and false negativity, which might be the result of a different platform analysis. Nevertheless, the six-mRNA signature model could well distinguish patients with different survival risks (log-rank test, *P* < 0.05). These results indicated that the novel signature had an important clinical value.

Studies have revealed important roles in cancer development for the six mRNAs of the signature model. For example, FRMD5 regulates cell motility by binding integrin β5 subunit and Rho-associated, coiled-coil containing protein kinase 1 (ROCK1)[27]. A recent study found that FRMD5 was a novel direct target of β-catenin/TCF7L2 complex. The accumulation of β-catenin and subsequent transactivation of TCF7L2 could deregulate the canonical Wnt signaling pathway and played an important role in human tumorigenesis [28]. The PCMT1 gene encodes a repair enzyme protein, which prevents Bax-induced apoptosis in neuronal cells and can be used as a potential therapeutic target for hepatocellular carcinoma [29]. PCMT1 was verified using the Western blot analysis to cross-react with the antisera of A5, and a protein antigen of A5 with significant immunotherapeutic effects on S180 sarcoma was successfully created by the induction of antibodies targeting for PCMT1 [30]. PDGFA promotes proliferation and self-renewal in glioblastoma stem cells [31]. It is upregulated in cholangiocarcinoma and significantly correlated with the status and clinical stage of cholangiocarcinoma. It can serve as a potential diagnostic and prognostic marker for cholangiocarcinoma [32]. Meanwhile, the functional enrichment analysis showed that PDGFA was involved in MAPK signaling pathway, pathways in cancer, and transcriptional dysregulation in cancer. TMC8 plays an important role in the transmembrane channel-like domain [33, 34], and its mutation is associated with high-risk HPV infection and HNSCC survival risk [33]. YIPF4 is a new cell-binding molecule of papillomavirus E5 [35], which is involved in regulating membrane dynamics in the endomembrane system [36]. ZNF324B is suggested to be involved in transcriptional regulation [37]. Also, the correlation of these six mRNAs was determined, and it was found that FRMD5 was significantly positively associated with PDGFA (*P* < 1e-10), and PCMT1 was significantly negatively correlated with ZNF324B (*P* < 1e-10), consistent with previous results that high expression levels of FRMD5, PCMT1, and PDGFA, but low expression levels of ZNF324B, were related to a higher risk. However, no studies have linked these genes to HNSCC prognosis. Nevertheless, the proposed signature model is a potential biomarker with the capacity to predict mortality hazard in HNSCC compared with selected existing biomarkers. It is helpful in improving the prognosis of HNSCC. However, the clinical application and effects of the six mRNAs in HNSCC prognosis need to be further evaluated. Related experiments may help further verifying the roles of these mRNAs in HNSCC prognosis and provide new insights into their mechanisms of action and functions in relation to HNSCC survival.

In conclusion, a six-mRNA signature that could be used as a prognostic factor for patient survival was identified in this study by analyzing high-throughput transcriptome sequencing data. Also, its practical value was confirmed in patients of different ages, genders, clinical stages, smoking history, and HPV status, as well as in the microarray dataset. Although further studies are needed to confirm the findings, the signature may be used as a potential biomarker for predicting patient survival and is of high clinical value.

## MATERIALS AND METHODS

### Gene expression data in HNSCC tissues from the TCGA dataset

The RNA-seq data of patients with HNSCC were downloaded from the TCGA dataset (https://tcga-data.nci.nih.gov/tcga/), and IlluminaHiSeqV2 data as well as related clinical information for a total of 519 patients were obtained. After excluding the samples without survival information, 500 samples that contained the expression data of 26,760 mRNA genes and transcripts were included in this study, with the mRNA expression level set as the Fragments Per Kilobase of transcript per Million mapped reads (FPKM) value. These samples in TCGA came from all over the world, instead of a single area, and all these samples were submitted into TCGA in batches and operated by different researchers according to the time sequentially. These 500 samples were stored in 18 batches. The first 6 batches contained 241 samples, nearly half of all the samples, and were considered the training set; the remaining 259 samples (12 batches) formed the testing set. The training set was used to identify the mRNAs associated with HNSCC prognosis and build the related model. The testing set was used to verify the accuracy of model prediction, thus determining the clinical predictive value of the model.

### Statistical analysis

In this study, OS was defined as the time from patient’s first diagnosis to death or last follow-up. As Li et al. reported [38], first Log2 conversion was used to normalize the RNA-seq data. Then, a univariate Cox regression analysis was employed to determine the genes significantly associated with HNSCC patient survival time according to the corresponding expression levels in the training set [12, 39, 40]. The Bioconductor Limma package in R language was used to determine differentially expressed genes between patients with long-term (>36 months) and short-term (<12 months) survival. Considering the fact that not all genes were expressed in HNSCC samples, genes with the capacity of protein coding and expressing in at least half of the HNSCC tissues were selected. A multivariate Cox regression analysis was performed to further screen the factors associated with patient survival. A proportional hazards model was constructed to generate a risk scoring formula, which helped determine a risk score for each patient. The patients were divided into low-risk and high-risk groups based on their scores. Then, the Kaplan-Meier analysis with log-rank test was used to evaluate the survival rates of patients in different risk groups [6]. Finally, ROC curves were used to assess the sensitivity and specificity of the risk assessment model in predicting survival in the testing set [41]. Enumeration data were presented as a percentage, and the chi-square test was used for comparison. For all tests, a *P* value <0.05 was considered statistically significant.

## SUPPLEMENTARY MATERIALS FIGURES AND TABLES




