# Supplementary material for: A six-mRNA signature model for the prognosis of head and neck squamous cell carcinoma
Source: Oncotarget. 2017 Oct 10;8(55):94528–38. doi: 10.18632/oncotarget.21786 (PMC5706893; doi:10.18632/oncotarget.21786)
Supplement: Supplementary file 1 [file oncotarget-08-94528-s001.pdf]

A six-mRNA signature model for the prognosis of head and neck squamous cell carcinoma

SUPPLEMENTARY MATERIALS

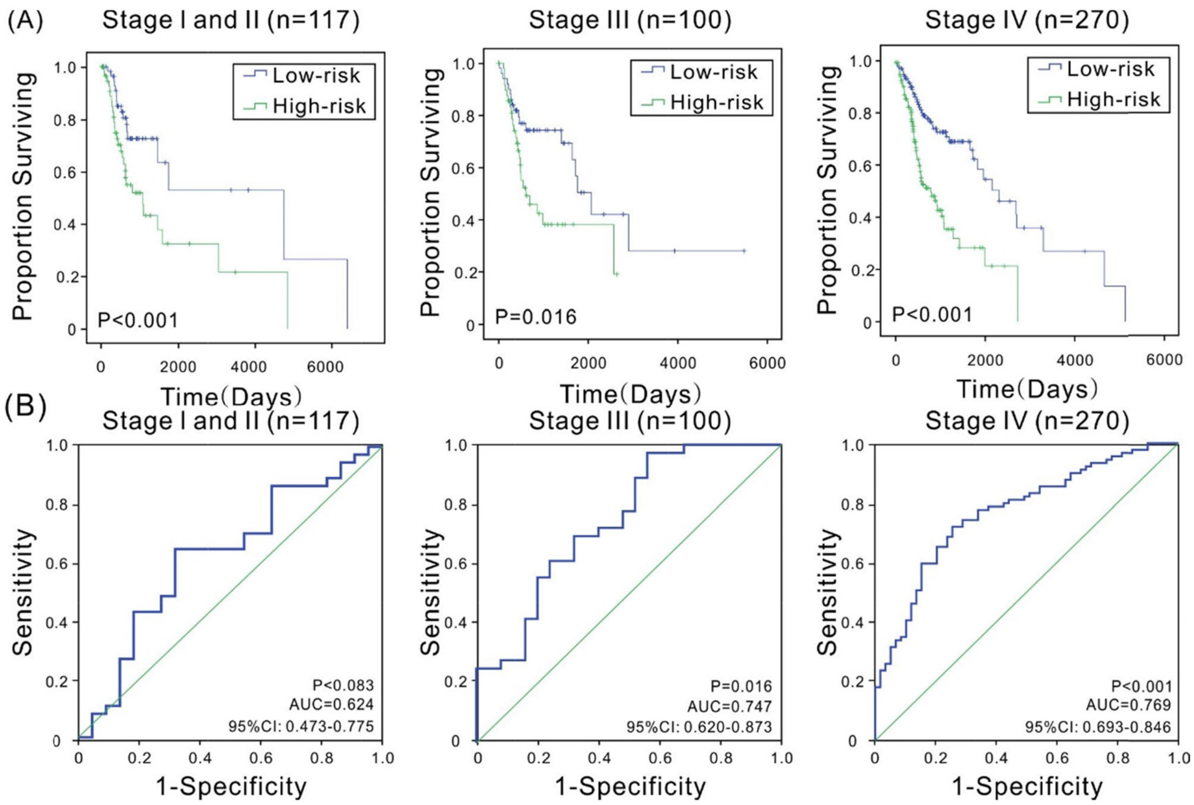

Supplementary Figure 1: Kaplan-Meier estimates of the overall survival of patients with different clinical stage. (A) Kaplan-Meier estimates of the overall survival of patients with different stage; **(B)** ROC curves show the sensitivity and specificity of the six-mRNA signature in predicting the patient overall survival.

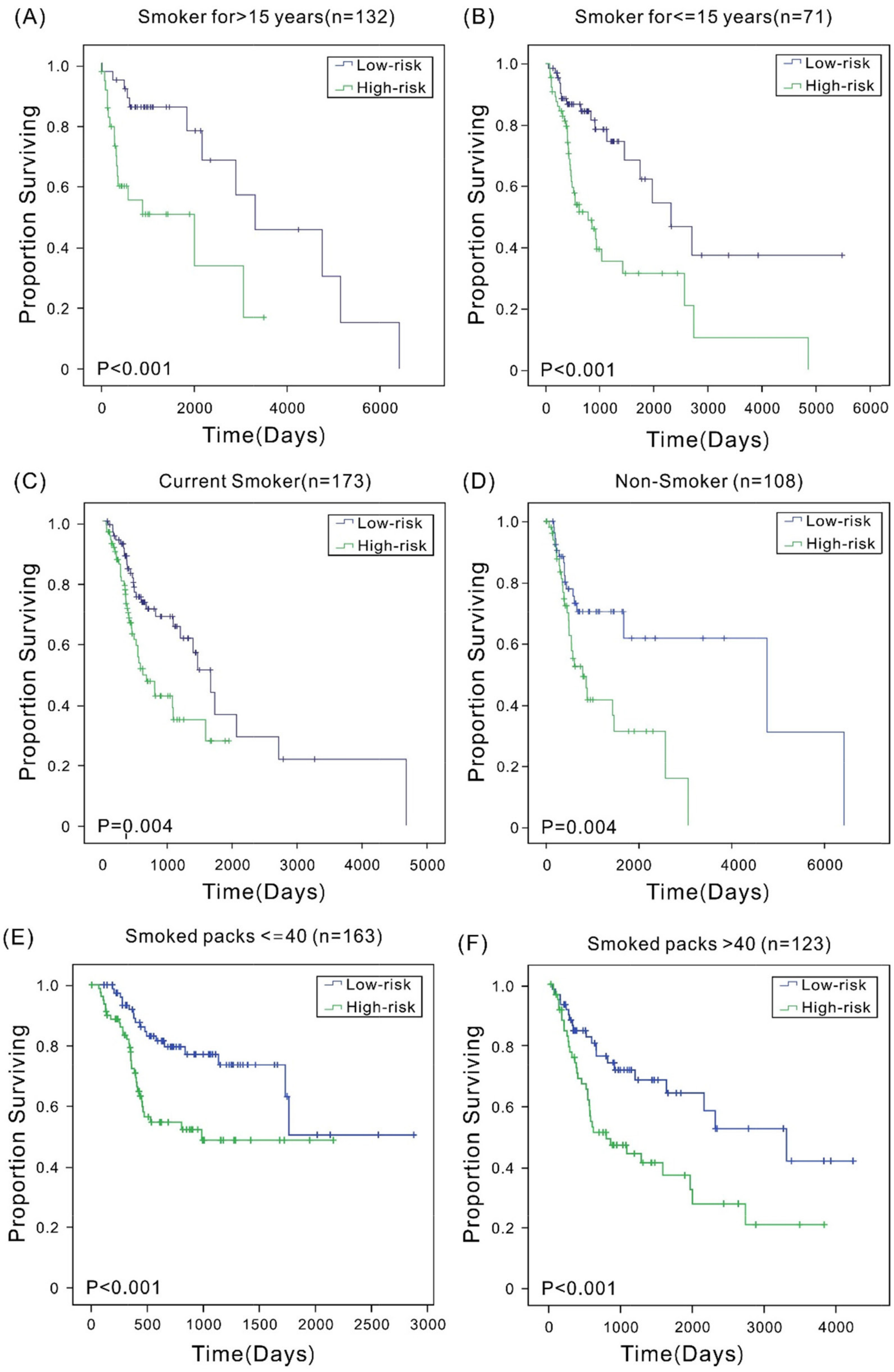

Supplementary Figure 2: Kaplan-Meier estimates of the overall survival of HNSCC patients with different smoking history.

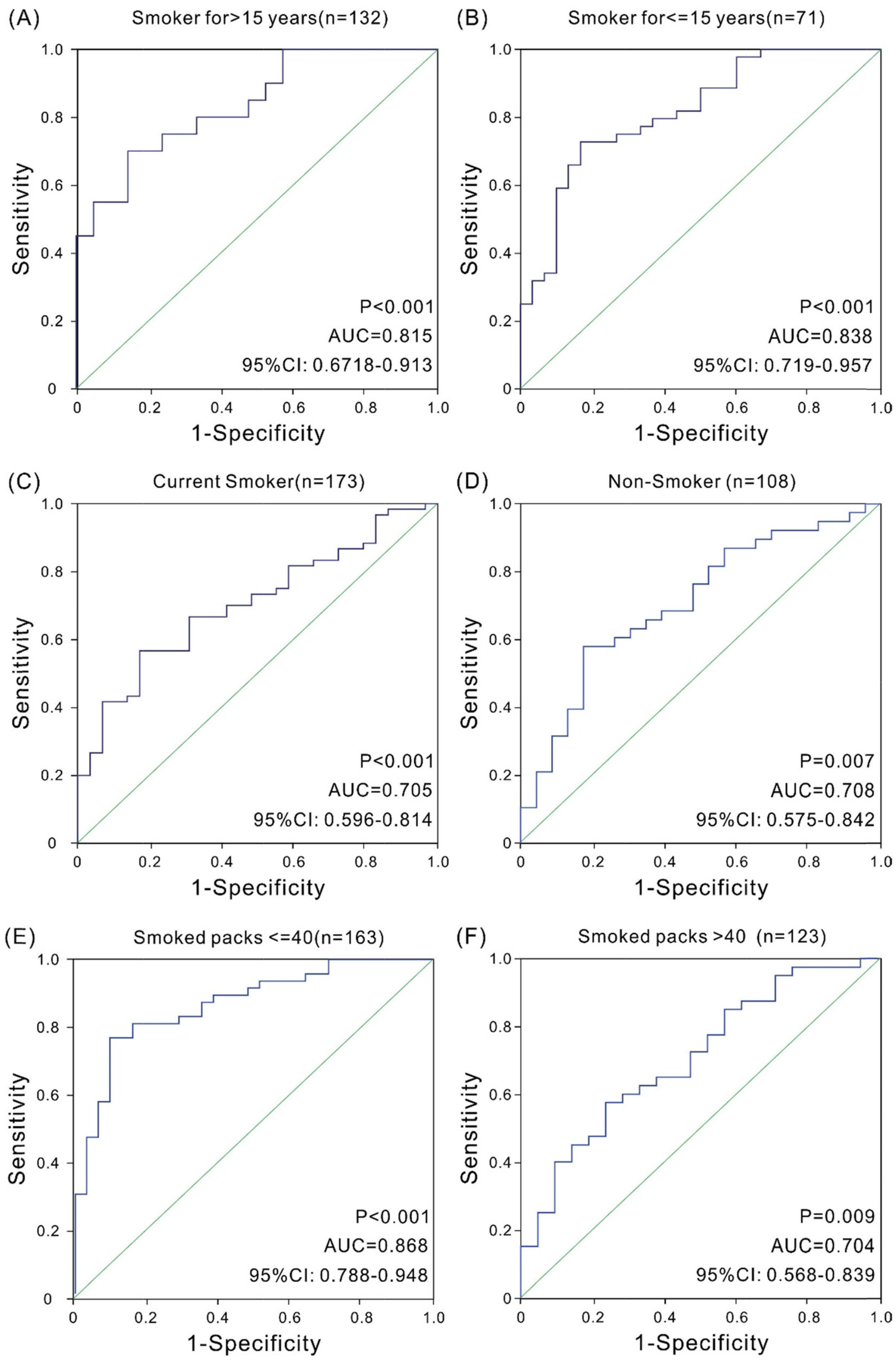

Supplementary Figure 3: ROC analysis of sensitivity and specificity using the six-mRNA signature in predicting the overall survival of HNSCC patients with different smoking history.

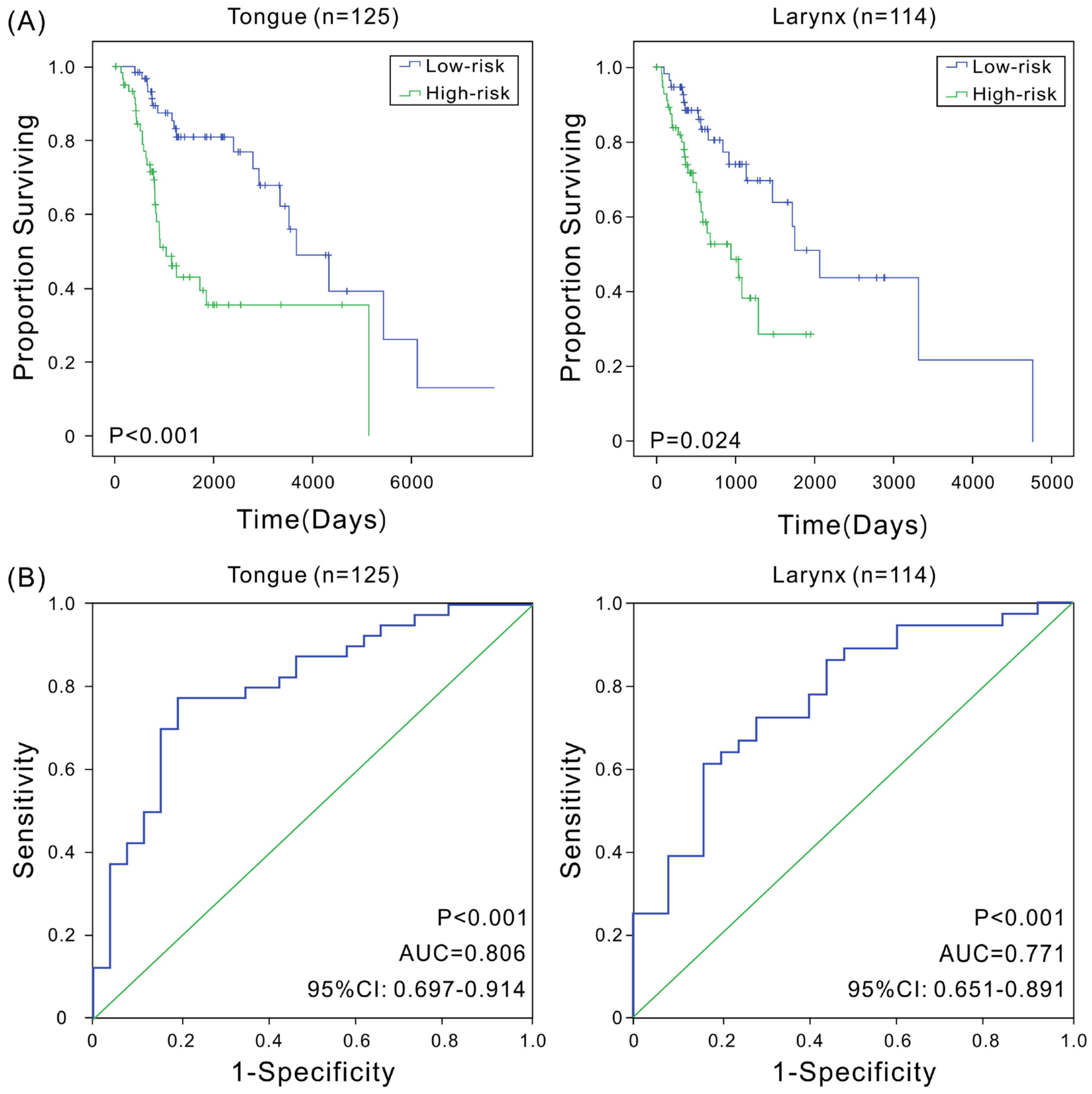

Supplementary Figure 4: Kaplan-Meier estimates of the overall survival of patients with different tumor tissue site (Tongue and Larynx). **(A)** Kaplan-Meier estimates of the overall survival of patients; **(B)** ROC curves show the sensitivity and specificity of the six-mRNA signature in predicting the patient overall survival.

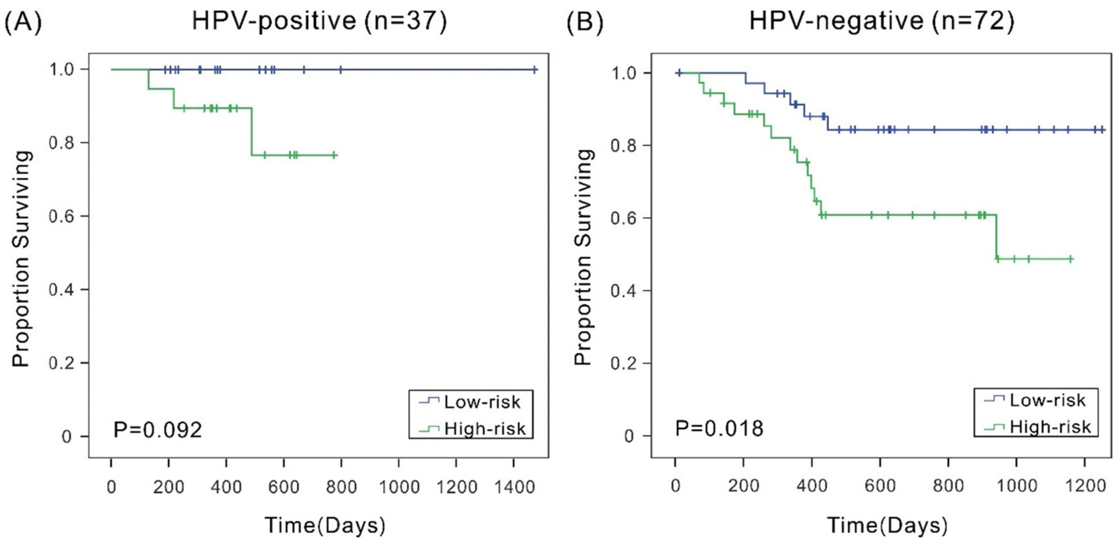

Supplementary Figure 5: Kaplan-Meier survival curves show correlation between the six-mRNA signature and overall survival in HPV-positive HNSCC patients **(A)** and HPV-negative HNSCC patients **(B).**

Supplementary Table 1: Sample list and information of HNSCC patients from TCGA database

See Supplementary File 1

Supplementary Table 2: Univariate Cox regression analysis with a significance level of 0.001 reveals a significant relation between mRNA expression and survival time, and the t test with differential expression between patients with longer OS and those with shorter OS time

See Supplementary File 1

Supplementary Table 3: Kaplan-Meier and ROC analyses of the three-lncRNA signature and 10 known biomarkers

Biomarker	AUC	*P*-value	95% CI of AUC	*P*-value[a]
Six-mRNA signature	0.745	<0.001	0.65-0.84	<0.001
TP53	0.552	0.369	0.44-0.66	0.329
PTK2	0.513	0.816	0.40-0.62	0.251
SEC62	0.596	0.096	0.49-0.71	0.235
MALAT1	0.520	0.730	0.41-0.63	0.245
HOTAIR	0.568	0.240	0.46-0.68	0.71
LCE5A	0.541	0.481	0.43-0.65	0.011
EIF4E	0.557	0.321	0.44-0.67	0.241
SOX4	0.550	0.386	0.44-0.66	0.164
MDM2	0.538	0.905	0.40-0.62	0.164
ANO1	0.538	0.513	0.43-0.65	0.205

a. log-rank test.
