# Supplementary material for: A six-mRNA signature model for the prognosis of head and neck squamous cell carcinoma
Source: Oncotarget. 2017 Oct 10;8(55):94528–38. doi: 10.18632/oncotarget.21786 (PMC5706893; doi:10.18632/oncotarget.21786)
Supplement: Supplementary file 2 [file oncotarget-08-94528-s002.docx]

**Table S1** Sample list and information of HNSCC patients from TCGA database.

| **TCGA_ID** | **OS status^a^** | **OS time (days)** | **Gender** | **Age** | **Stage** | **Tumor tissue** | **HPV status** | **Smoker** | **Packs smoked per year** |
| --- | --- | --- | --- | --- | --- | --- | --- | --- | --- |
| TCGA-4P-AA8J | 0 | 102 | MALE | 66 | Stage IVA | Tongue | Negative | [Unknown] | NA |
| TCGA-BA-4074 | 1 | 462 | MALE | 69 | Stage IVA | Tongue | NA | Current smoker | 51 |
| TCGA-BA-4075 | 1 | 283 | MALE | 49 | Stage IVA | Tongue | NA | Current smoker | 30 |
| TCGA-BA-4076 | 1 | 415 | MALE | 39 | Stage IVA | Larynx | NA | Current smoker | 30 |
| TCGA-BA-4077 | 1 | 1134 | FEMALE | 45 | Stage IVB | Base of Tongue | NA | Current reformed smoker for≤ 15 years | 30 |
| TCGA-BA-4078 | 1 | 276 | MALE | 83 | Stage IVA | Larynx | NA | Current reformed smoker for≤15 years | 75 |
| TCGA-BA-5149 | 1 | 806 | MALE | 47 | Stage IVA | Floor of Mouth | NA | Current smoker | 60 |
| TCGA-BA-5151 | 0 | 722 | MALE | 72 | Stage IVA | Buccal mucosa | NA | Current reformed smoker for> 15 years | 20 |
| TCGA-BA-5152 | 0 | 1288 | MALE | 56 | Stage IVA | Alveolar Ridge | NA | Lifelong Non-smoker | NA |
| TCGA-BA-5153 | 1 | 1762 | MALE | 51 | Stage III | Tonsil | NA | Lifelong Non-smoker | NA |
| TCGA-BA-5555 | 0 | 520 | MALE | 54 | Stage IVA | Larynx | NA | Current reformed smoker for≤15 years | 62 |
| TCGA-BA-5556 | 0 | 725 | FEMALE | 58 | Stage II | Floor of Mouth | NA | Current reformed smoker for≤15 years | 60 |
| TCGA-BA-5557 | 0 | 623 | FEMALE | 41 | Stage IVA | Tongue | NA | Lifelong Non-smoker | NA |
| TCGA-BA-5558 | 0 | 1636 | MALE | 65 | Stage IVA | Hard Palate | NA | Lifelong Non-smoker | NA |
| TCGA-BA-5559 | 0 | 1747 | MALE | 71 | Stage IVA | Tonsil | NA | Lifelong Non-smoker | NA |
| TCGA-BA-6868 | 1 | 472 | MALE | 53 | Stage IVB | Larynx | NA | Current reformed smoker for≤15 years | 60 |
| TCGA-BA-6869 | 0 | 644 | MALE | 62 | Stage III | Larynx | NA | Current smoker | 46 |
| TCGA-BA-6870 | 1 | 451 | FEMALE | 60 | Stage IVC | Larynx | NA | Current smoker | 40 |
| TCGA-BA-6871 | 1 | 108 | MALE | 75 | Stage IVA | Base of Tongue | NA | Current reformed smoker for≤15 years | 180 |
| TCGA-BA-6872 | 1 | 384 | MALE | 47 | Stage IVA | Floor of Mouth | NA | Current smoker | 40 |
| TCGA-BA-6873 | 0 | 113 | MALE | 28 | Stage IVA | Tongue | NA | Current smoker | 1 |
| TCGA-BA-7269 | 0 | 236 | MALE | 61 | Stage III | Tongue | NA | Current smoker | 46 |
| TCGA-BA-A4IF | 0 | 895 | MALE | 59 | Stage IVA | Oropharynx | Negative | Current reformed smoker for≤15 years | 40 |
| TCGA-BA-A4IG | 0 | 855 | MALE | 77 | Stage IVA | Base of Tongue | NA | Current reformed smoker for> 15 years | NA |
| TCGA-BA-A4IH | 0 | 622 | MALE | 57 | Stage IVA | Tonsil | Positive | Current reformed smoker for≤15 years | 20 |
| TCGA-BA-A4II | 0 | 918 | MALE | 46 | Stage I | Oropharynx | Not Evaluated | Current reformed smoker for≤15 years | 25 |
| TCGA-BA-A6D8 | 0 | 850 | MALE | 59 | Stage IVA | Floor of Mouth | Negative | Current reformed smoker for≤15 years | 40 |
| TCGA-BA-A6DA | 0 | 351 | FEMALE | 41 | Stage IVA | Larynx | Positive | Current smoker | 25 |
| TCGA-BA-A6DB | 0 | 216 | FEMALE | 24 | Stage II | Tongue | Negative | Lifelong Non-smoker | NA |
| TCGA-BA-A6DD | 1 | 173 | MALE | 44 | Stage IVA | Floor of Mouth | Negative | Current smoker | 30 |
| TCGA-BA-A6DE | 0 | 440 | FEMALE | 70 | Stage III | Tongue | Negative | Current smoker | 60 |
| TCGA-BA-A6DG | 1 | 69 | MALE | 49 | Stage IVA | Tongue | Negative | Current reformed smoker for> 15 years | 20 |
| TCGA-BA-A6DI | 1 | 336 | MALE | 62 | Stage III | Larynx | Negative | Current smoker | 40 |
| TCGA-BA-A6DJ | 1 | 407 | MALE | 62 | Stage IVA | Alveolar Ridge | Negative | Current reformed smoker for≤15 years | 40 |
| TCGA-BA-A6DL | 0 | 623 | MALE | 59 | Stage III | Oropharynx | Negative | Current smoker | 45 |
| TCGA-BA-A8YP | 0 | 142 | MALE | 50 | Stage IVB | Oropharynx | Negative | Current smoker | 14 |
| TCGA-BB-4217 | 0 | 187 | MALE | 68 | Stage IVA | Larynx | NA | Current smoker | 60 |
| TCGA-BB-4223 | 0 | 2878 | MALE | 48 | Stage IVA | Tonsil | NA | Lifelong Non-smoker | NA |
| TCGA-BB-4224 | 0 | 278 | MALE | 52 | Stage III | Tongue | NA | Lifelong Non-smoker | NA |
| TCGA-BB-4225 | 0 | 140 | MALE | 73 | Stage IVA | Base of Tongue | NA | Current reformed smoker for≤15 years | 1.5 |
| TCGA-BB-4227 | 0 | 133 | MALE | 66 | Stage IVA | Hypopharynx | NA | Current reformed smoker for> 15 years | 25 |
| TCGA-BB-4228 | 0 | 558 | MALE | 50 | Stage II | Base of Tongue | Positive | Current smoker | 102.5 |
| TCGA-BB-7861 | 0 | 310 | MALE | 56 | Stage III | Base of Tongue | Positive | Current smoker | NA |
| TCGA-BB-7862 | 0 | 536 | MALE | 67 | Stage IVA | Larynx | NA | Current reformed smoker for≤15 years | 90 |
| TCGA-BB-7863 | 0 | 423 | FEMALE | 43 | Stage III | Tongue | NA | Lifelong Non-smoker | NA |
| TCGA-BB-7864 | 0 | 687 | MALE | 61 | Stage IVA | Larynx | NA | Current reformed smoker for≤15 years | 80 |
| TCGA-BB-7866 | 0 | 638 | MALE | 40 | Stage IVA | Tonsil | Positive | Current reformed smoker for≤15 years | NA |
| TCGA-BB-7870 | 0 | 1153 | MALE | 58 | Stage IVA | Larynx | NA | Current smoker | 40 |
| TCGA-BB-7871 | 0 | 428 | FEMALE | 64 | Stage IVA | Base of Tongue | NA | Current smoker | 5.1 |
| TCGA-BB-7872 | 0 | 436 | MALE | 63 | Stage IVA | Tongue | Positive | Lifelong Non-smoker | NA |
| TCGA-BB-8596 | 0 | 2161 | FEMALE | 69 | Stage IVA | Hypopharynx | Not Evaluated | Current reformed smoker for≤15 years | 50 |
| TCGA-BB-8601 | 0 | 624 | MALE | 84 | Stage II | Floor of Mouth | Not Evaluated | Current reformed smoker for≤15 years | NA |
| TCGA-C9-A47Z | 1 | 191 | FEMALE | 72 | Stage III | Tongue | NA | Lifelong Non-smoker | NA |
| TCGA-C9-A480 | 0 | 4 | FEMALE | 45 | Stage III | Tongue | NA | Lifelong Non-smoker | NA |
| TCGA-CN-4722 | 0 | 799 | FEMALE | 61 | Stage II | Larynx | NA | Current smoker | NA |
| TCGA-CN-4723 | 0 | 721 | MALE | 67 | Stage IVA | Larynx | NA | Current reformed smoker for≤15 years | NA |
| TCGA-CN-4725 | 0 | 1157 | MALE | 60 | Stage II | Tongue | Negative | Lifelong Non-smoker | NA |
| TCGA-CN-4726 | 1 | 142 | MALE | 68 | Stage IVA | Buccal mucosa | Negative | Current smoker | 12.5 |
| TCGA-CN-4727 | 0 | 1230 | MALE | 56 | Stage IVA | Larynx | Negative | Current reformed smoker for≤15 years | 80 |
| TCGA-CN-4728 | 0 | 1151 | MALE | 56 | Stage IVA | Oral Cavity | Negative | Current smoker | 40 |
| TCGA-CN-4729 | 0 | 392 | MALE | 73 | Stage IVA | Oral Cavity | NA | Current reformed smoker for≤15 years | 35 |
| TCGA-CN-4730 | 0 | 817 | MALE | 62 | Stage IVA | Floor of Mouth | NA | Current smoker | 47 |
| TCGA-CN-4731 | 0 | 993 | FEMALE | 63 | Stage IVA | Buccal mucosa | Negative | Current reformed smoker for≤15 years | 60 |
| TCGA-CN-4733 | 0 | 642 | MALE | 61 | Stage I | Tongue | Negative | Lifelong Non-smoker | NA |
| TCGA-CN-4734 | 0 | 758 | MALE | 70 | Stage II | Buccal mucosa | Negative | Current reformed smoker for≤15 years | NA |
| TCGA-CN-4735 | 0 | 1250 | MALE | 52 | Stage IVA | Larynx | Negative | Current smoker | 120 |
| TCGA-CN-4736 | 1 | 395 | FEMALE | 70 | Stage II | Tongue | NA | Current reformed smoker for≤15 years | 45 |
| TCGA-CN-4737 | 0 | 625 | MALE | 19 | Stage II | Tongue | Negative | Lifelong Non-smoker | NA |
| TCGA-CN-4738 | 1 | 436 | MALE | 53 | Stage IVA | Larynx | NA | Current smoker | 72 |
| TCGA-CN-4739 | 0 | 1330 | MALE | 71 | Stage IVA | Larynx | NA | Current reformed smoker for≤15 years | 82.5 |
| TCGA-CN-4740 | 1 | 839 | FEMALE | 79 | Stage IVA | Oral Cavity | NA | Current reformed smoker for≤15 years | 12 |
| TCGA-CN-4741 | 0 | 1268 | MALE | 75 | Stage IVA | Alveolar Ridge | NA | Current reformed smoker for≤15 years | 45 |
| TCGA-CN-4742 | 1 | 397 | FEMALE | 48 | Stage IVA | Tongue | Negative | Current smoker | 20 |
| TCGA-CN-5355 | 0 | 1007 | MALE | 64 | Stage IVA | Larynx | NA | Current smoker | 40 |
| TCGA-CN-5356 | 0 | 1045 | MALE | 56 | Stage III | Larynx | NA | Current smoker | NA |
| TCGA-CN-5358 | 1 | 261 | MALE | 60 | Stage III | Floor of Mouth | Negative | Current reformed smoker for≤15 years | 45 |
| TCGA-CN-5359 | 1 | 377 | MALE | 59 | Stage IVA | Floor of Mouth | Negative | Current smoker | 29 |
| TCGA-CN-5360 | 0 | 1081 | MALE | 68 | Stage IVA | Larynx | NA | Current smoker | 50 |
| TCGA-CN-5361 | 0 | 1681 | MALE | 80 | NA | Larynx | NA | Current smoker | 25 |
| TCGA-CN-5363 | 1 | 253 | MALE | 48 | Stage IVB | Larynx | NA | Current smoker | 15 |
| TCGA-CN-5364 | 1 | 493 | MALE | 55 | Stage IVA | Floor of Mouth | NA | Current smoker | NA |
| TCGA-CN-5365 | 1 | 351 | MALE | 38 | Stage IVC | Tonsil | NA | Current smoker | 26 |
| TCGA-CN-5366 | 1 | 360 | MALE | 51 | Stage IVA | Hypopharynx | NA | Current smoker | 80 |
| TCGA-CN-5367 | 1 | 352 | FEMALE | 60 | Stage IVA | Tongue | NA | Current reformed smoker for≤15 years | 40 |
| TCGA-CN-5369 | 1 | 380 | FEMALE | 90 | Stage IVA | Hard Palate | NA | Current smoker | NA |
| TCGA-CN-5370 | 1 | 259 | MALE | 78 | Stage II | Tongue | Negative | Current reformed smoker for≤15 years | NA |
| TCGA-CN-5373 | 0 | 683 | FEMALE | 55 | Stage II | Floor of Mouth | Negative | Current reformed smoker for≤15 years | 60 |
| TCGA-CN-5374 | 1 | 1732 | FEMALE | 56 | Stage IVA | Tonsil | NA | Current smoker | 30 |
| TCGA-CN-6010 | 0 | 630 | MALE | 53 | Stage IVA | Larynx | Negative | Current smoker | 67.5 |
| TCGA-CN-6011 | 0 | 933 | MALE | 57 | Stage IVA | Alveolar Ridge | NA | Current reformed smoker for≤15 years | 40 |
| TCGA-CN-6012 | 0 | 629 | MALE | 66 | Stage III | Larynx | Negative | Current smoker | 100 |
| TCGA-CN-6013 | 0 | 628 | MALE | 56 | Stage IVA | Alveolar Ridge | Negative | Lifelong Non-smoker | NA |
| TCGA-CN-6016 | 0 | 594 | MALE | 64 | Stage IVA | Floor of Mouth | Negative | Current smoker | 50 |
| TCGA-CN-6017 | 0 | 629 | MALE | 55 | Stage III | Tongue | Negative | Current reformed smoker for> 15 years | 5 |
| TCGA-CN-6018 | 1 | 580 | FEMALE | 85 | Stage IVA | Oral Cavity | NA | Lifelong Non-smoker | NA |
| TCGA-CN-6019 | 0 | 432 | MALE | 61 | Stage IVA | Tongue | Negative | Current reformed smoker for≤15 years | 20 |
| TCGA-CN-6020 | 1 | 205 | MALE | 58 | Stage IVA | Oral Cavity | Negative | Current smoker | NA |
| TCGA-CN-6021 | 1 | 276 | FEMALE | 63 | Stage III | Larynx | NA | Current reformed smoker for≤15 years | 60 |
| TCGA-CN-6022 | 1 | 281 | MALE | 49 | Stage IVA | Larynx | Negative | Current smoker | NA |
| TCGA-CN-6023 | 0 | 1110 | MALE | 73 | Stage IVA | Larynx | Negative | Current reformed smoker for> 15 years | 35 |
| TCGA-CN-6024 | 1 | 337 | MALE | 66 | Stage IVA | Tongue | Negative | Current smoker | 150 |
| TCGA-CN-6988 | 0 | 318 | MALE | 47 | Stage IVA | Larynx | Negative | Current reformed smoker for≤15 years | 40 |
| TCGA-CN-6989 | 0 | 611 | MALE | 64 | Stage IVA | Larynx | Negative | Current smoker | 50 |
| TCGA-CN-6992 | 0 | 394 | MALE | 61 | Stage IVA | Larynx | Negative | Current smoker | 90 |
| TCGA-CN-6994 | 0 | 735 | MALE | 67 | Stage IVA | Oral Cavity | NA | Current reformed smoker for> 15 years | 12 |
| TCGA-CN-6995 | 1 | 112 | MALE | 78 | Stage IVA | Floor of Mouth | NA | Current smoker | NA |
| TCGA-CN-6996 | 1 | 530 | FEMALE | 58 | Stage IVA | Tongue | NA | Lifelong Non-smoker | NA |
| TCGA-CN-6997 | 0 | 429 | MALE | 66 | Stage IVA | Larynx | Negative | Current reformed smoker for≤15 years | 80 |
| TCGA-CN-6998 | 1 | 357 | MALE | 53 | Stage IVA | Tongue | Negative | Current smoker | 40 |
| TCGA-CN-A497 | 0 | 1065 | MALE | 63 | Stage IVA | Larynx | Negative | Current reformed smoker for≤15 years | NA |
| TCGA-CN-A498 | 0 | 443 | FEMALE | 61 | Stage II | Tongue | Not Evaluated | Current reformed smoker for≤15 years | 40 |
| TCGA-CN-A499 | 0 | 535 | FEMALE | 60 | Stage I | Tonsil | Positive | Lifelong Non-smoker | NA |
| TCGA-CN-A49A | 0 | 391 | MALE | 60 | Stage IVA | Alveolar Ridge | Not Evaluated | Lifelong Non-smoker | NA |
| TCGA-CN-A49B | 0 | 904 | MALE | 71 | Stage III | Larynx | Negative | Current reformed smoker for≤15 years | 40 |
| TCGA-CN-A49C | 0 | 645 | MALE | 67 | Stage IVA | Tonsil | Positive | Current reformed smoker for> 15 years | 45 |
| TCGA-CN-A63T | 0 | 225 | MALE | 60 | Stage IVA | Larynx | Negative | Current smoker | 46 |
| TCGA-CN-A63U | 0 | 526 | MALE | 50 | Stage III | Larynx | Negative | Current smoker | 25 |
| TCGA-CN-A63W | 0 | 348 | FEMALE | 48 | Stage IVA | Larynx | Negative | Current smoker | NA |
| TCGA-CN-A641 | 0 | 367 | MALE | 47 | Stage IVA | Larynx | Positive | Current smoker | 15 |
| TCGA-CN-A642 | 1 | 82 | MALE | 57 | Stage IVA | Tongue | Negative | Current reformed smoker for≤15 years | 5 |
| TCGA-CN-A6UY | 0 | 307 | MALE | 57 | Stage IVA | Base of Tongue | Positive | Current reformed smoker for≤15 years | 40 |
| TCGA-CN-A6V1 | 0 | 223 | MALE | 59 | Stage IVA | Tonsil | Positive | Lifelong Non-smoker | NA |
| TCGA-CN-A6V3 | 0 | 298 | MALE | 61 | Stage IVA | Larynx | NA | Current reformed smoker for≤15 years | 40 |
| TCGA-CN-A6V6 | 0 | 234 | MALE | 59 | Stage IVA | Base of Tongue | Positive | Current reformed smoker for≤15 years | 90 |
| TCGA-CN-A6V7 | 0 | 188 | MALE | 40 | Stage IVA | Tonsil | Positive | Lifelong Non-smoker | NA |
| TCGA-CQ-5323 | 0 | 1466 | MALE | 82 | Stage III | Alveolar Ridge | NA | Current reformed smoker for> 15 years | 45 |
| TCGA-CQ-5324 | 0 | 1207 | MALE | 59 | Stage IVA | Floor of Mouth | NA | Current reformed smoker for≤15 years | 43 |
| TCGA-CQ-5325 | 1 | 654 | MALE | 65 | Stage I | Tongue | NA | Current reformed smoker for≤15 years | 49 |
| TCGA-CQ-5326 | 1 | 89 | MALE | 67 | Stage IVA | Alveolar Ridge | NA | Current reformed smoker for≤15 years | 40 |
| TCGA-CQ-5327 | 0 | 1660 | FEMALE | 61 | Stage IVA | Tongue | NA | Lifelong Non-smoker | NA |
| TCGA-CQ-5329 | 0 | 1422 | FEMALE | 46 | Stage III | Tongue | NA | Lifelong Non-smoker | NA |
| TCGA-CQ-5330 | 0 | 1393 | FEMALE | 69 | Stage III | Tongue | NA | Current reformed smoker for> 15 years | 21 |
| TCGA-CQ-5331 | 0 | 1399 | FEMALE | 73 | Stage IVA | Hard Palate | NA | Current reformed smoker for> 15 years | 15 |
| TCGA-CQ-5332 | 1 | 317 | MALE | 87 | Stage III | Floor of Mouth | NA | Current reformed smoker for≤15 years | 90 |
| TCGA-CQ-5333 | 1 | 341 | MALE | 74 | Stage I | Tongue | NA | Current reformed smoker for> 15 years | 37 |
| TCGA-CQ-5334 | 1 | 129 | MALE | 87 | Stage IVB | Buccal mucosa | NA | Current reformed smoker for> 15 years | NA |
| TCGA-CQ-6218 | 0 | 1253 | FEMALE | 52 | Stage III | Floor of Mouth | NA | Current reformed smoker for≤15 years | 30 |
| TCGA-CQ-6219 | 1 | 479 | FEMALE | 50 | Stage IVA | Tongue | NA | Current smoker | 35 |
| TCGA-CQ-6220 | 1 | 985 | MALE | 69 | Stage III | Buccal mucosa | NA | Lifelong Non-smoker | NA |
| TCGA-CQ-6221 | 0 | 1000 | MALE | 79 | Stage II | Tongue | NA | Current reformed smoker for> 15 years | 25 |
| TCGA-CQ-6222 | 0 | 2016 | MALE | 63 | Stage IVA | Tongue | NA | Current reformed smoker for> 15 years | 10 |
| TCGA-CQ-6223 | 0 | 1057 | MALE | 69 | Stage IVA | Alveolar Ridge | NA | Current reformed smoker for> 15 years | 22 |
| TCGA-CQ-6224 | 0 | 1721 | MALE | 52 | Stage II | Tongue | NA | Current reformed smoker for≤15 years | 35 |
| TCGA-CQ-6225 | 1 | 403 | MALE | 65 | Stage II | Tongue | NA | Current reformed smoker for≤15 years | NA |
| TCGA-CQ-6227 | 1 | 129 | MALE | 77 | Stage III | Oral Cavity | NA | Current reformed smoker for> 15 years | 20 |
| TCGA-CQ-6228 | 1 | 456 | FEMALE | 71 | Stage III | Floor of Mouth | NA | Current smoker | 50 |
| TCGA-CQ-6229 | 0 | 1179 | MALE | 61 | Stage III | Tongue | NA | Lifelong Non-smoker | NA |
| TCGA-CQ-7063 | 0 | 2133 | FEMALE | 59 | NA | Hard Palate | Not Evaluated | Current reformed smoker for> 15 years | 1.25 |
| TCGA-CQ-7065 | 0 | 1007 | MALE | 40 | Stage II | Tongue | NA | Lifelong Non-smoker | NA |
| TCGA-CQ-7067 | 0 | 509 | FEMALE | 75 | Stage I | Tongue | NA | NA | NA |
| TCGA-CQ-7068 | 0 | 1309 | FEMALE | 80 | Stage II | Floor of Mouth | NA | Lifelong Non-smoker | NA |
| TCGA-CQ-7069 | 0 | 1274 | FEMALE | 77 | Stage II | Alveolar Ridge | Not Evaluated | Lifelong Non-smoker | NA |
| TCGA-CQ-7071 | 0 | 877 | FEMALE | 76 | Stage IVA | Oral Cavity | Not Evaluated | Lifelong Non-smoker | NA |
| TCGA-CQ-7072 | 0 | 1950 | MALE | 51 | Stage IVA | Floor of Mouth | Not Evaluated | Current smoker | 40 |
| TCGA-CQ-A4C6 | 0 | 1353 | MALE | 63 | Stage II | Buccal mucosa | Not Evaluated | Current reformed smoker for≤15 years | 3 |
| TCGA-CQ-A4C7 | 1 | 353 | MALE | 88 | Stage II | Floor of Mouth | Not Evaluated | Current reformed smoker for> 15 years | 25 |
| TCGA-CQ-A4C9 | 0 | 707 | MALE | 56 | NA | Floor of Mouth | Not Evaluated | Current smoker | 6 |
| TCGA-CQ-A4CB | 0 | 893 | MALE | 59 | Stage II | Floor of Mouth | Not Evaluated | Current smoker | 75 |
| TCGA-CQ-A4CD | 0 | 998 | MALE | 69 | Stage IVB | Oral Cavity | Not Evaluated | Lifelong Non-smoker | NA |
| TCGA-CQ-A4CE | 0 | 785 | FEMALE | 76 | Stage II | Tongue | Not Evaluated | Lifelong Non-smoker | NA |
| TCGA-CQ-A4CG | 1 | 430 | FEMALE | 78 | Stage II | Buccal mucosa | Not Evaluated | Current reformed smoker for≤15 years | 70 |
| TCGA-CQ-A4CH | 0 | 303 | MALE | 58 | Stage IVA | Tongue | Not Evaluated | Lifelong Non-smoker | NA |
| TCGA-CQ-A4CI | 0 | 950 | MALE | 73 | NA | Buccal mucosa | Not Evaluated | Current reformed smoker for≤15 years | 40 |
| TCGA-CR-5243 | 0 | 2562 | MALE | 51 | Stage IVA | Tonsil | NA | Lifelong Non-smoker | NA |
| TCGA-CR-5247 | 0 | 358 | MALE | 48 | Stage III | Tonsil | NA | Current smoker | 105 |
| TCGA-CR-5248 | 0 | 1663 | MALE | 53 | Stage IVA | Tonsil | NA | Current smoker | 35 |
| TCGA-CR-5249 | 0 | 1152 | FEMALE | 35 | Stage II | Tonsil | NA | Lifelong Non-smoker | NA |
| TCGA-CR-5250 | 0 | 799 | MALE | 71 | Stage II | Base of Tongue | Positive | Current smoker | 30 |
| TCGA-CR-6467 | 0 | 1777 | MALE | 59 | Stage IVA | Tonsil | NA | Current reformed smoker for≤15 years | 15 |
| TCGA-CR-6470 | 0 | 1521 | MALE | 38 | Stage IVA | Tonsil | NA | Lifelong Non-smoker | NA |
| TCGA-CR-6471 | 1 | 1202 | MALE | 58 | Stage IVA | Oral Cavity | NA | Current smoker | 40 |
| TCGA-CR-6472 | 0 | 1050 | MALE | 59 | Stage IVB | Base of Tongue | NA | Lifelong Non-smoker | NA |
| TCGA-CR-6473 | 0 | 1125 | MALE | 68 | Stage IVA | Hypopharynx | NA | Current reformed smoker for≤15 years | 50 |
| TCGA-CR-6474 | 1 | 564 | MALE | 51 | Stage IVA | Larynx | NA | Current smoker | 74 |
| TCGA-CR-6477 | 0 | 514 | FEMALE | 56 | Stage IVA | Base of Tongue | Negative | Current reformed smoker for> 15 years | NA |
| TCGA-CR-6478 | 1 | 183 | FEMALE | 66 | Stage IVA | Tonsil | NA | Current reformed smoker for≤15 years | NA |
| TCGA-CR-6480 | 0 | 362 | MALE | 53 | Stage IVA | Tonsil | Positive | Lifelong Non-smoker | NA |
| TCGA-CR-6481 | 0 | 311 | MALE | 47 | Stage IVA | Tonsil | Positive | Lifelong Non-smoker | NA |
| TCGA-CR-6482 | 0 | 345 | MALE | 62 | Stage IVA | Tonsil | Positive | Current reformed smoker for≤15 years | 31 |
| TCGA-CR-6484 | 0 | 354 | FEMALE | 67 | Stage IVA | Oral Cavity | Negative | Lifelong Non-smoker | NA |
| TCGA-CR-6487 | 0 | 234 | MALE | 50 | Stage II | Tonsil | Positive | Current reformed smoker for≤15 years | 11 |
| TCGA-CR-6488 | 0 | 379 | FEMALE | 68 | Stage II | Tongue | NA | Lifelong Non-smoker | NA |
| TCGA-CR-6491 | 0 | 350 | MALE | 60 | Stage IVA | Floor of Mouth | Negative | Current smoker | 67.5 |
| TCGA-CR-6492 | 0 | 479 | MALE | 78 | Stage IVA | Hard Palate | Negative | Current smoker | 50 |
| TCGA-CR-6493 | 1 | 282 | MALE | 69 | Stage IVA | Tongue | NA | Current reformed smoker for> 15 years | 15 |
| TCGA-CR-7364 | 0 | 1435 | MALE | 66 | Stage III | Larynx | NA | Current smoker | 150 |
| TCGA-CR-7365 | 0 | 1191 | MALE | 60 | Stage IVA | Oral Cavity | NA | Current smoker | NA |
| TCGA-CR-7367 | 0 | 1440 | MALE | 52 | Stage IVB | Oral Cavity | NA | Current smoker | 45 |
| TCGA-CR-7368 | 0 | 1245 | MALE | 54 | Stage IVA | Oral Cavity | NA | Current reformed smoker for≤15 years | 57 |
| TCGA-CR-7369 | 1 | 1090 | MALE | 59 | Stage IVA | Oral Cavity | NA | Current smoker | 48 |
| TCGA-CR-7370 | 0 | 105 | FEMALE | 72 | Stage II | Larynx | NA | Current reformed smoker for≤15 years | 112 |
| TCGA-CR-7371 | 1 | 94 | FEMALE | 45 | Stage III | Larynx | NA | Current smoker | 60 |
| TCGA-CR-7372 | 0 | 759 | MALE | 45 | Stage II | Tongue | Negative | Lifelong Non-smoker | NA |
| TCGA-CR-7373 | 0 | 889 | MALE | 66 | Stage IVA | Oral Cavity | Negative | Current reformed smoker for> 15 years | 90 |
| TCGA-CR-7374 | 0 | 30 | FEMALE | 67 | Stage II | Larynx | NA | Current smoker | 45 |
| TCGA-CR-7376 | 0 | 972 | MALE | 83 | Stage II | Oral Cavity | Negative | Current reformed smoker for> 15 years | 54 |
| TCGA-CR-7377 | 1 | 279 | MALE | 58 | Stage IVA | Oral Cavity | NA | Current smoker | 80 |
| TCGA-CR-7379 | 0 | 1036 | FEMALE | 78 | Stage IVA | Oral Cavity | Negative | Current reformed smoker for> 15 years | 0.71 |
| TCGA-CR-7380 | 1 | 606 | MALE | 58 | Stage III | Oral Cavity | NA | Lifelong Non-smoker | NA |
| TCGA-CR-7382 | 0 | 796 | MALE | 49 | Stage IVA | Tongue | NA | Lifelong Non-smoker | NA |
| TCGA-CR-7383 | 1 | 521 | FEMALE | 79 | Stage I | Tonsil | NA | Current smoker | 110 |
| TCGA-CR-7385 | 0 | 997 | MALE | 42 | Stage IVA | Tonsil | NA | Current reformed smoker for> 15 years | 0.0169 |
| TCGA-CR-7386 | 0 | 1430 | MALE | 69 | Stage IVA | Oral Cavity | NA | Current reformed smoker for> 15 years | 54 |
| TCGA-CR-7388 | 1 | 823 | FEMALE | 70 | Stage IVA | Larynx | NA | Current smoker | 110 |
| TCGA-CR-7389 | 0 | 392 | MALE | 55 | Stage III | Larynx | NA | Current smoker | 34 |
| TCGA-CR-7390 | 0 | 907 | MALE | 67 | Stage III | Tongue | Negative | Current smoker | 80 |
| TCGA-CR-7391 | 0 | 913 | FEMALE | 36 | Stage I | Tongue | Negative | Current reformed smoker for> 15 years | NA |
| TCGA-CR-7392 | 0 | 946 | FEMALE | 67 | Stage IVA | Tongue | Negative | Current reformed smoker for> 15 years | 30 |
| TCGA-CR-7393 | 0 | 908 | MALE | 26 | Stage I | Tongue | Negative | Lifelong Non-smoker | NA |
| TCGA-CR-7394 | 0 | 898 | MALE | 70 | Stage IVA | Tongue | Negative | Current smoker | 60 |
| TCGA-CR-7395 | 0 | 930 | FEMALE | 80 | Stage II | Oral Cavity | Negative | Current reformed smoker for> 15 years | 60 |
| TCGA-CR-7397 | 0 | 216 | MALE | 44 | Stage IVA | Tongue | NA | Current reformed smoker for≤15 years | NA |
| TCGA-CR-7398 | 0 | 156 | FEMALE | 53 | Stage II | Larynx | NA | Current smoker | 40 |
| TCGA-CR-7399 | 0 | 181 | FEMALE | 60 | Stage IVA | Larynx | NA | Current smoker | 135 |
| TCGA-CR-7401 | 0 | 1077 | MALE | 64 | Stage I | Tongue | NA | Current reformed smoker for≤15 years | 66 |
| TCGA-CR-7402 | 0 | 911 | MALE | 68 | Stage III | Larynx | NA | Current smoker | 108 |
| TCGA-CR-7404 | 0 | 1472 | MALE | 53 | Stage IVA | Tonsil | Positive | Lifelong Non-smoker | NA |
| TCGA-CV-5430 | 0 | 4241 | MALE | 61 | Stage IVA | Larynx | NA | Current reformed smoker for> 15 years | NA |
| TCGA-CV-5431 | 1 | 522 | MALE | 73 | Stage IVA | Larynx | NA | Current reformed smoker for≤15 years | NA |
| TCGA-CV-5432 | 0 | 3930 | MALE | 68 | Stage III | Larynx | NA | Current reformed smoker for≤15 years | NA |
| TCGA-CV-5434 | 1 | 3314 | MALE | 60 | Stage IVA | Larynx | NA | Current reformed smoker for> 15 years | NA |
| TCGA-CV-5435 | 1 | 2319 | MALE | 57 | Stage IVA | Larynx | NA | Current reformed smoker for≤15 years | NA |
| TCGA-CV-5436 | 1 | 584 | MALE | 65 | Stage IVA | Floor of Mouth | NA | Current smoker | NA |
| TCGA-CV-5439 | 1 | 546 | MALE | 62 | Stage II | Base of Tongue | NA | Current smoker | NA |
| TCGA-CV-5440 | 0 | 3270 | MALE | 52 | Stage IVA | Larynx | NA | Current smoker | NA |
| TCGA-CV-5441 | 0 | 2886 | MALE | 58 | Stage IVA | Larynx | NA | Current reformed smoker for≤15 years | NA |
| TCGA-CV-5442 | 0 | 2327 | FEMALE | 76 | Stage IVA | Hard Palate | NA | Current reformed smoker for≤15 years | NA |
| TCGA-CV-5443 | 0 | 2784 | MALE | 63 | Stage III | Larynx | NA | Current smoker | NA |
| TCGA-CV-5444 | 0 | 2437 | MALE | 64 | Stage IVA | Larynx | NA | Current reformed smoker for≤15 years | NA |
| TCGA-CV-5966 | 1 | 545 | FEMALE | 63 | Stage IVA | Oral Cavity | NA | Current reformed smoker for≤15 years | NA |
| TCGA-CV-5970 | 1 | 406 | MALE | 59 | Stage IVA | Tongue | NA | Current reformed smoker for≤15 years | NA |
| TCGA-CV-5971 | 0 | 701 | MALE | 60 | Stage IVA | Tongue | NA | Current smoker | NA |
| TCGA-CV-5973 | 0 | 2641 | FEMALE | 62 | Stage III | Tongue | NA | Lifelong Non-smoker | NA |
| TCGA-CV-5976 | 0 | 1478 | MALE | 50 | Stage III | Tongue | NA | Current reformed smoker for≤15 years | NA |
| TCGA-CV-5977 | 0 | 1840 | MALE | 66 | Stage III | Tongue | NA | Current reformed smoker for≤15 years | NA |
| TCGA-CV-5978 | 1 | 215 | FEMALE | 53 | Stage IVB | Larynx | NA | Current smoker | NA |
| TCGA-CV-5979 | 0 | 1315 | MALE | 26 | Stage III | Tongue | NA | Lifelong Non-smoker | NA |
| TCGA-CV-6003 | 0 | 1665 | FEMALE | 50 | Stage II | Tongue | NA | Lifelong Non-smoker | NA |
| TCGA-CV-6433 | 0 | 641 | MALE | 57 | Stage II | Tongue | NA | Current smoker | NA |
| TCGA-CV-6436 | 0 | 1899 | MALE | 62 | Stage III | Tongue | NA | Current reformed smoker for> 15 years | 9.5 |
| TCGA-CV-6441 | 1 | 292 | MALE | 60 | Stage III | Tongue | NA | Current reformed smoker for≤15 years | 38 |
| TCGA-CV-6933 | 1 | 2741 | MALE | 53 | Stage IVA | Tongue | NA | Current reformed smoker for≤15 years | 60 |
| TCGA-CV-6934 | 1 | 65 | FEMALE | 66 | Stage IVA | Tongue | NA | Current smoker | 75 |
| TCGA-CV-6935 | 1 | 295 | MALE | 67 | Stage III | Larynx | NA | Current smoker | NA |
| TCGA-CV-6936 | 1 | 166 | MALE | 68 | Stage IVA | Floor of Mouth | NA | Current smoker | NA |
| TCGA-CV-6937 | 1 | 624 | MALE | 71 | Stage II | Oral Cavity | NA | Current reformed smoker for≤15 years | NA |
| TCGA-CV-6938 | 1 | 144 | MALE | 87 | Stage II | Oral Cavity | NA | Current reformed smoker for> 15 years | NA |
| TCGA-CV-6939 | 1 | 666 | MALE | 60 | Stage IVA | Tongue | NA | Lifelong Non-smoker | NA |
| TCGA-CV-6940 | 1 | 804 | FEMALE | 80 | Stage I | Buccal mucosa | NA | Current smoker | 50 |
| TCGA-CV-6941 | 1 | 342 | MALE | 51 | Stage III | Tongue | NA | Current smoker | 60 |
| TCGA-CV-6942 | 0 | 3835 | FEMALE | 73 | Stage II | Oral Cavity | NA | Lifelong Non-smoker | NA |
| TCGA-CV-6943 | 1 | 602 | MALE | 74 | Stage III | Base of Tongue | NA | Current reformed smoker for> 15 years | NA |
| TCGA-CV-6945 | 1 | 366 | MALE | 41 | Stage IVA | Tongue | NA | Current smoker | 75 |
| TCGA-CV-6948 | 1 | 1289 | FEMALE | 79 | Stage IVA | Floor of Mouth | NA | NA | NA |
| TCGA-CV-6950 | 1 | 459 | MALE | 64 | Stage IVA | Base of Tongue | NA | Current reformed smoker for≤15 years | NA |
| TCGA-CV-6951 | 1 | 915 | MALE | 57 | Stage IVA | Tongue | NA | Current reformed smoker for≤15 years | NA |
| TCGA-CV-6952 | 1 | 185 | FEMALE | 65 | Stage III | Tongue | NA | Current reformed smoker for≤15 years | 30 |
| TCGA-CV-6953 | 1 | 1641 | FEMALE | 80 | Stage III | Floor of Mouth | NA | Lifelong Non-smoker | NA |
| TCGA-CV-6954 | 1 | 2002 | MALE | 59 | Stage IVA | Tongue | NA | Current reformed smoker for> 15 years | NA |
| TCGA-CV-6955 | 1 | 334 | FEMALE | 74 | Stage II | Oral Cavity | NA | Lifelong Non-smoker | NA |
| TCGA-CV-6956 | 1 | 217 | MALE | 67 | Stage IVA | Tongue | NA | Current smoker | NA |
| TCGA-CV-6959 | 1 | 256 | MALE | 48 | Stage III | Tongue | NA | Lifelong Non-smoker | NA |
| TCGA-CV-6960 | 1 | 862 | MALE | 49 | Stage III | Oral Cavity | NA | Current reformed smoker for≤15 years | NA |
| TCGA-CV-6961 | 1 | 76 | MALE | 61 | Stage II | Tongue | NA | Current reformed smoker for≤15 years | 66 |
| TCGA-CV-6962 | 1 | 126 | MALE | 65 | Stage III | Larynx | NA | Current reformed smoker for≤15 years | NA |
| TCGA-CV-7089 | 1 | 1972 | MALE | 74 | Stage IVA | Larynx | NA | Current reformed smoker for≤15 years | NA |
| TCGA-CV-7090 | 0 | 3837 | MALE | 39 | Stage II | Oral Cavity | NA | NA | NA |
| TCGA-CV-7091 | 0 | 3381 | MALE | 54 | Stage I | Oral Cavity | NA | Current reformed smoker for≤15 years | NA |
| TCGA-CV-7095 | 1 | 572 | FEMALE | 87 | Stage IVA | Oral Cavity | NA | Current reformed smoker for> 15 years | 40 |
| TCGA-CV-7097 | 1 | 385 | MALE | 53 | Stage III | Oral Cavity | NA | Current reformed smoker for≤15 years | 64 |
| TCGA-CV-7099 | 1 | 243 | FEMALE | 85 | Stage II | Oral Cavity | NA | Current reformed smoker for> 15 years | NA |
| TCGA-CV-7100 | 1 | 274 | MALE | 66 | Stage II | Oral Cavity | NA | Current reformed smoker for> 15 years | NA |
| TCGA-CV-7101 | 1 | 160 | MALE | 80 | Stage II | Larynx | NA | Current smoker | NA |
| TCGA-CV-7102 | 1 | 56 | FEMALE | 76 | Stage III | Floor of Mouth | NA | Current reformed smoker for≤15 years | NA |
| TCGA-CV-7103 | 1 | 1591 | MALE | 49 | Stage II | Tongue | NA | Current smoker | NA |
| TCGA-CV-7104 | 1 | 393 | FEMALE | 61 | Stage IVA | Tongue | NA | Lifelong Non-smoker | NA |
| TCGA-CV-7177 | 1 | 663 | FEMALE | 82 | Stage I | Larynx | NA | NA | NA |
| TCGA-CV-7178 | 1 | 2166 | FEMALE | 64 | Stage IVA | Oral Cavity | NA | Current reformed smoker for> 15 years | NA |
| TCGA-CV-7180 | 1 | 327 | MALE | 34 | Stage II | Tongue | NA | Current reformed smoker for> 15 years | NA |
| TCGA-CV-7183 | 0 | 3497 | MALE | 53 | Stage II | Oral Cavity | NA | Current reformed smoker for> 15 years | NA |
| TCGA-CV-7235 | 0 | 2347 | MALE | 67 | Stage III | Floor of Mouth | NA | Current reformed smoker for> 15 years | 97.5 |
| TCGA-CV-7236 | 1 | 144 | FEMALE | 77 | Stage IVA | Tongue | NA | NA | NA |
| TCGA-CV-7238 | 0 | 1444 | FEMALE | 69 | Stage II | Tongue | NA | Lifelong Non-smoker | NA |
| TCGA-CV-7242 | 0 | 1095 | FEMALE | 60 | Stage III | Larynx | NA | Current reformed smoker for> 15 years | 40 |
| TCGA-CV-7243 | 0 | 954 | MALE | 50 | Stage II | Tongue | NA | Lifelong Non-smoker | NA |
| TCGA-CV-7245 | 0 | 797 | MALE | 62 | Stage III | Larynx | NA | Current reformed smoker for≤15 years | 60 |
| TCGA-CV-7247 | 1 | 577 | MALE | 55 | Stage II | Larynx | NA | Lifelong Non-smoker | NA |
| TCGA-CV-7248 | 1 | 521 | FEMALE | 63 | Stage IVA | Larynx | NA | Current smoker | 30 |
| TCGA-CV-7250 | 1 | 2900 | MALE | 64 | Stage III | Larynx | NA | Current reformed smoker for> 15 years | 60 |
| TCGA-CV-7252 | 1 | 151 | FEMALE | 62 | Stage III | Oral Cavity | NA | NA | NA |
| TCGA-CV-7253 | 1 | 361 | MALE | 58 | Stage II | Oral Cavity | NA | NA | NA |
| TCGA-CV-7254 | 1 | 1459 | MALE | 55 | Stage II | Oral Cavity | NA | Current reformed smoker for≤15 years | 40 |
| TCGA-CV-7255 | 1 | 64 | FEMALE | 32 | Stage II | Tongue | NA | Lifelong Non-smoker | NA |
| TCGA-CV-7261 | 0 | 1099 | MALE | 57 | Stage III | Larynx | NA | Current smoker | 41 |
| TCGA-CV-7263 | 1 | 560 | MALE | 64 | Stage II | Oral Cavity | NA | Current reformed smoker for> 15 years | 60 |
| TCGA-CV-7406 | 1 | 1748 | MALE | 49 | Stage II | Base of Tongue | NA | Current reformed smoker for≤15 years | 9 |
| TCGA-CV-7407 | 1 | 1081 | FEMALE | 67 | Stage II | Floor of Mouth | NA | Lifelong Non-smoker | NA |
| TCGA-CV-7409 | 1 | 543 | MALE | 43 | Stage IVB | Oral Cavity | NA | NA | NA |
| TCGA-CV-7410 | 1 | 6417 | MALE | 61 | Stage II | Larynx | NA | Current reformed smoker for> 15 years | 38 |
| TCGA-CV-7411 | 1 | 2717 | FEMALE | 64 | Stage IVA | Oral Cavity | NA | Current smoker | NA |
| TCGA-CV-7413 | 1 | 294 | FEMALE | 74 | Stage II | Oral Cavity | NA | NA | NA |
| TCGA-CV-7414 | 1 | 14 | MALE | 78 | Stage III | Oral Cavity | NA | Current reformed smoker for> 15 years | NA |
| TCGA-CV-7415 | 1 | 695 | MALE | 60 | Stage III | Larynx | NA | NA | NA |
| TCGA-CV-7416 | 1 | 763 | FEMALE | 29 | Stage IVA | Oral Cavity | NA | Lifelong Non-smoker | NA |
| TCGA-CV-7418 | 1 | 789 | MALE | 62 | Stage IVA | Larynx | NA | Current reformed smoker for≤15 years | 60 |
| TCGA-CV-7421 | 1 | 2 | MALE | 76 | Stage IVA | Larynx | NA | Current reformed smoker for> 15 years | NA |
| TCGA-CV-7422 | 1 | 1037 | FEMALE | 60 | Stage IVA | Larynx | NA | Current reformed smoker for≤15 years | 80 |
| TCGA-CV-7423 | 1 | 3059 | MALE | 65 | Stage II | Oral Cavity | NA | Current reformed smoker for> 15 years | NA |
| TCGA-CV-7424 | 1 | 453 | MALE | 67 | Stage IVA | Larynx | NA | Current reformed smoker for≤15 years | 50 |
| TCGA-CV-7425 | 1 | 1718 | FEMALE | 77 | Stage III | Oral Cavity | NA | Lifelong Non-smoker | NA |
| TCGA-CV-7427 | 1 | 4760 | FEMALE | 73 | Stage II | Oral Cavity | NA | Current reformed smoker for> 15 years | 25 |
| TCGA-CV-7428 | 1 | 1671 | MALE | 47 | Stage IVA | Oral Cavity | NA | Current smoker | 4 |
| TCGA-CV-7429 | 1 | 107 | MALE | 55 | Stage III | Oral Cavity | NA | Current reformed smoker for≤15 years | NA |
| TCGA-CV-7430 | 1 | 495 | MALE | 56 | Stage III | Larynx | NA | Current reformed smoker for≤15 years | 40 |
| TCGA-CV-7432 | 1 | 2570 | MALE | 79 | Stage III | Oral Cavity | NA | Current reformed smoker for≤15 years | 61 |
| TCGA-CV-7433 | 1 | 601 | MALE | 49 | Stage IVA | Larynx | NA | Current smoker | 16 |
| TCGA-CV-7434 | 1 | 218 | MALE | 64 | Stage III | Oral Cavity | NA | Current reformed smoker for≤15 years | 80 |
| TCGA-CV-7435 | 1 | 4680 | FEMALE | 57 | Stage IVA | Oral Cavity | NA | Current smoker | NA |
| TCGA-CV-7437 | 1 | 506 | MALE | 77 | Stage II | Larynx | NA | Lifelong Non-smoker | NA |
| TCGA-CV-7438 | 1 | 194 | FEMALE | 87 | Stage II | Tongue | NA | Lifelong Non-smoker | NA |
| TCGA-CV-7440 | 1 | 675 | MALE | 38 | Stage II | Larynx | NA | Current smoker | 21 |
| TCGA-CV-7446 | 1 | 1093 | MALE | 66 | Stage II | Tongue | NA | Current smoker | 86 |
| TCGA-CV-7568 | 1 | 927 | FEMALE | 48 | Stage IVA | Oral Cavity | NA | Current reformed smoker for≤15 years | 35 |
| TCGA-CV-A45O | 0 | 851 | MALE | 57 | Stage III | Alveolar Ridge | NA | Current reformed smoker for> 15 years | NA |
| TCGA-CV-A45P | 0 | 639 | FEMALE | 82 | Stage II | Tongue | NA | Lifelong Non-smoker | NA |
| TCGA-CV-A45Q | 1 | 5152 | FEMALE | 69 | Stage IVC | Oral Cavity | NA | Current reformed smoker for> 15 years | NA |
| TCGA-CV-A45R | 0 | 5480 | MALE | 46 | Stage III | Tongue | NA | Current reformed smoker for≤15 years | NA |
| TCGA-CV-A45T | 1 | 4856 | FEMALE | 64 | Stage II | Tongue | NA | Current reformed smoker for≤15 years | NA |
| TCGA-CV-A45U | 1 | 1079 | MALE | 59 | Stage IVA | Oral Cavity | NA | Current smoker | NA |
| TCGA-CV-A45V | 1 | 32 | FEMALE | 87 | Stage IVA | Oral Cavity | NA | Lifelong Non-smoker | NA |
| TCGA-CV-A45W | 1 | 1398 | MALE | 75 | Stage III | Larynx | NA | Current smoker | NA |
| TCGA-CV-A45X | 1 | 198 | MALE | 47 | Stage IVA | Floor of Mouth | NA | Current smoker | NA |
| TCGA-CV-A45Y | 1 | 2703 | MALE | 61 | Stage IVA | Larynx | NA | Current reformed smoker for≤15 years | 39 |
| TCGA-CV-A45Z | 1 | 1466 | MALE | 75 | Stage II | Larynx | NA | Current smoker | 40 |
| TCGA-CV-A460 | 1 | 1838 | MALE | 72 | Stage IVA | Larynx | NA | Current reformed smoker for> 15 years | 51 |
| TCGA-CV-A461 | 1 | 2064 | MALE | 65 | Stage III | Larynx | NA | Current smoker | NA |
| TCGA-CV-A463 | 1 | 23 | FEMALE | 82 | Stage IVA | Floor of Mouth | NA | Lifelong Non-smoker | NA |
| TCGA-CV-A464 | 0 | 1722 | MALE | 48 | Stage IVA | Buccal mucosa | NA | Current reformed smoker for≤15 years | 21 |
| TCGA-CV-A465 | 1 | 215 | MALE | 24 | Stage II | Tongue | NA | Lifelong Non-smoker | NA |
| TCGA-CV-A468 | 1 | 464 | MALE | 42 | Stage III | Lip | NA | Current smoker | 25 |
| TCGA-CV-A6JD | 1 | 182 | FEMALE | 82 | Stage IVA | Floor of Mouth | NA | Current reformed smoker for> 15 years | 10 |
| TCGA-CV-A6JE | 0 | 1075 | MALE | 78 | Stage II | Oral Cavity | NA | Current reformed smoker for≤15 years | NA |
| TCGA-CV-A6JM | 1 | 194 | MALE | 85 | Stage IVA | Hypopharynx | NA | Lifelong Non-smoker | NA |
| TCGA-CV-A6JN | 0 | 906 | MALE | 53 | Stage II | Oral Cavity | NA | Current smoker | 54 |
| TCGA-CV-A6JO | 1 | 197 | MALE | 69 | Stage IVA | Tongue | NA | Current reformed smoker for≤15 years | 74 |
| TCGA-CV-A6JT | 0 | 670 | MALE | 65 | Stage II | Tongue | NA | Lifelong Non-smoker | NA |
| TCGA-CV-A6JU | 0 | 110 | FEMALE | 61 | Stage IVA | Tongue | NA | Current smoker | 81 |
| TCGA-CV-A6JY | 0 | 646 | MALE | 69 | Stage IVA | Oral Cavity | NA | Current smoker | NA |
| TCGA-CV-A6JZ | 0 | 714 | MALE | 68 | Stage IVA | Oral Cavity | NA | Current smoker | 53 |
| TCGA-CV-A6K0 | 0 | 606 | MALE | 58 | Stage II | Tongue | NA | Lifelong Non-smoker | NA |
| TCGA-CV-A6K1 | 0 | 685 | MALE | 65 | Stage IVA | Larynx | NA | Current reformed smoker for≤15 years | 43 |
| TCGA-CV-A6K2 | 1 | 317 | MALE | 79 | Stage II | Oral Cavity | NA | Current reformed smoker for> 15 years | 14 |
| TCGA-CX-7082 | 0 | 317 | MALE | 82 | Stage II | Oral Cavity | NA | Current reformed smoker for≤15 years | 50 |
| TCGA-CX-7085 | 0 | 321 | FEMALE | 77 | Stage III | Tongue | NA | Current reformed smoker for> 15 years | 3 |
| TCGA-CX-7086 | 0 | 573 | MALE | 53 | Stage IVA | Floor of Mouth | NA | Current smoker | 35 |
| TCGA-CX-7219 | 0 | 1045 | MALE | 47 | Stage IVA | Floor of Mouth | NA | Current smoker | 20 |
| TCGA-D6-6515 | 1 | 403 | FEMALE | 82 | Stage II | Tongue | NA | Lifelong Non-smoker | NA |
| TCGA-D6-6516 | 0 | 773 | MALE | 69 | Stage III | Lip | NA | Current reformed smoker for≤15 years | 74 |
| TCGA-D6-6517 | 0 | 292 | MALE | 59 | Stage III | Larynx | NA | Current reformed smoker for≤15 years | 30 |
| TCGA-D6-6823 | 0 | 701 | MALE | 50 | Stage III | Tongue | NA | Current smoker | 60 |
| TCGA-D6-6824 | 0 | 77 | MALE | 61 | Stage IVA | Larynx | NA | Current smoker | 40 |
| TCGA-D6-6825 | 0 | 491 | MALE | 73 | Stage III | Tongue | NA | Current reformed smoker for≤15 years | 300 |
| TCGA-D6-6826 | 1 | 348 | FEMALE | 64 | Stage IVA | Larynx | NA | Lifelong Non-smoker | NA |
| TCGA-D6-6827 | 0 | 568 | FEMALE | 55 | Stage III | Lip | NA | Current smoker | 28 |
| TCGA-D6-8568 | 0 | 106 | MALE | 62 | Stage II | Larynx | Not Evaluated | Current smoker | 43 |
| TCGA-D6-8569 | 0 | 128 | MALE | 52 | Stage II | Tongue | Not Evaluated | Current reformed smoker for≤15 years | 43 |
| TCGA-D6-A4Z9 | 0 | 539 | MALE | 59 | Stage IVA | Tongue | Not Evaluated | Current reformed smoker for> 15 years | 39 |
| TCGA-D6-A4ZB | 0 | 376 | MALE | 61 | Stage III | Tongue | Not Evaluated | Current smoker | 41 |
| TCGA-D6-A6EK | 0 | 483 | MALE | 67 | Stage IVA | Larynx | Not Evaluated | Current reformed smoker for≤15 years | 72 |
| TCGA-D6-A6EM | 0 | 232 | FEMALE | 65 | Stage III | Tongue | Not Evaluated | Lifelong Non-smoker | NA |
| TCGA-D6-A6EO | 0 | 435 | MALE | 44 | Stage IVA | Floor of Mouth | Not Evaluated | Current Reformed Smoker, Duration Not Specified | NA |
| TCGA-D6-A6EP | 0 | 424 | MALE | 62 | Stage III | Hypopharynx | Not Evaluated | Current reformed smoker for> 15 years | 40 |
| TCGA-D6-A6EQ | 0 | 368 | MALE | 57 | Stage IVA | Larynx | Not Evaluated | Lifelong Non-smoker | NA |
| TCGA-D6-A6ES | 0 | 389 | MALE | 50 | Stage IVA | Larynx | Not Evaluated | [Unknown] | NA |
| TCGA-D6-A74Q | 0 | 338 | MALE | 67 | Stage IVA | Larynx | Not Evaluated | Current Reformed Smoker, Duration Not Specified | NA |
| TCGA-DQ-5624 | 0 | 1778 | FEMALE | 43 | Stage IVA | Tongue | NA | Lifelong Non-smoker | NA |
| TCGA-DQ-5625 | 0 | 1081 | FEMALE | 52 | Stage II | Tongue | NA | Current smoker | 33 |
| TCGA-DQ-5629 | 1 | 941 | MALE | 64 | Stage IVA | Larynx | Negative | Current reformed smoker for≤15 years | 63 |
| TCGA-DQ-5630 | 0 | 1030 | MALE | 73 | Stage III | Tongue | NA | Current reformed smoker for> 15 years | 5 |
| TCGA-DQ-5631 | 1 | 548 | MALE | 52 | Stage IVA | Tongue | NA | Current reformed smoker for≤15 years | 23 |
| TCGA-DQ-7588 | 1 | 427 | MALE | 66 | Stage III | Buccal mucosa | Negative | Current reformed smoker for≤15 years | 45 |
| TCGA-DQ-7589 | 0 | 575 | MALE | 70 | Stage IVC | Larynx | Negative | Current reformed smoker for≤15 years | 100 |
| TCGA-DQ-7590 | 0 | 775 | MALE | 51 | Stage IVA | Tonsil | Positive | Current reformed smoker for> 15 years | 0.5 |
| TCGA-DQ-7591 | 0 | 622 | MALE | 62 | Stage IVA | Base of Tongue | Positive | Current reformed smoker for> 15 years | 20 |
| TCGA-DQ-7592 | 0 | 299 | MALE | 57 | Stage IVA | Tongue | Negative | Current smoker | 68 |
| TCGA-DQ-7593 | 0 | 369 | MALE | 58 | Stage IVA | Base of Tongue | Positive | Current smoker | 20 |
| TCGA-DQ-7594 | 0 | 368 | MALE | 47 | Stage IVB | Base of Tongue | Positive | Lifelong Non-smoker | NA |
| TCGA-DQ-7595 | 0 | 324 | MALE | 53 | Stage II | Larynx | Positive | Current smoker | 80 |
| TCGA-DQ-7596 | 0 | 340 | MALE | 48 | Stage IVA | Tonsil | NA | Lifelong Non-smoker | NA |
| TCGA-F7-7848 | 0 | 1131 | MALE | 47 | Stage IVA | Larynx | NA | Current smoker | 20 |
| TCGA-F7-8298 | 0 | 490 | MALE | 58 | Stage I | Larynx | NA | Current reformed smoker for> 15 years | 26 |
| TCGA-F7-8489 | 0 | 658 | MALE | 48 | Stage II | Floor of Mouth | Unknown | Current smoker | 300 |
| TCGA-F7-A50G | 0 | 616 | MALE | 66 | NA | Tongue | Not Evaluated | Current smoker | 27 |
| TCGA-F7-A50I | 0 | 92 | MALE | 72 | NA | Larynx | Unknown | Current smoker | 0.9 |
| TCGA-F7-A50J | 0 | 585 | FEMALE | 67 | NA | Tongue | Not Evaluated | Current reformed smoker for≤15 years | 25.5 |
| TCGA-F7-A61S | 0 | 576 | MALE | 62 | NA | Tongue | Not Evaluated | Current reformed smoker for≤15 years | 11 |
| TCGA-F7-A61V | 0 | 384 | MALE | 54 | NA | Base of Tongue | Negative | Current smoker | 38 |
| TCGA-F7-A61W | 0 | 14 | MALE | 51 | NA | Tongue | Not Evaluated | Current smoker | 20 |
| TCGA-F7-A620 | 0 | 13 | MALE | 47 | NA | Base of Tongue | Not Evaluated | Current smoker | 30 |
| TCGA-F7-A623 | 0 | 616 | MALE | 70 | NA | Larynx | Not Evaluated | Current smoker | 42 |
| TCGA-F7-A624 | 0 | 378 | MALE | 73 | NA | Buccal mucosa | Not Evaluated | Lifelong Non-smoker | NA |
| TCGA-H7-7774 | 0 | 407 | FEMALE | 75 | Stage III | Oral Cavity | NA | Current reformed smoker for≤15 years | 55 |
| TCGA-H7-8501 | 0 | 461 | MALE | 54 | Stage IVA | Buccal mucosa | Unknown | Current smoker | NA |
| TCGA-H7-8502 | 0 | 458 | MALE | 50 | Stage IVA | Oral Cavity | Unknown | Current smoker | 40 |
| TCGA-H7-A6C4 | 0 | 414 | FEMALE | 35 | Stage III | Tongue | Negative | Current smoker | 9 |
| TCGA-H7-A76A | 0 | 206 | MALE | 57 | Stage IVA | Tonsil | Positive | Current reformed smoker for≤15 years | NA |
| TCGA-HD-7229 | 0 | 2 | MALE | 60 | Stage IVA | Larynx | NA | Current reformed smoker for> 15 years | NA |
| TCGA-HD-7753 | 0 | 5 | MALE | 62 | Stage II | Oropharynx | NA | Current smoker | 40 |
| TCGA-HD-7754 | 0 | 783 | MALE | 69 | Stage IVA | Tonsil | NA | Current reformed smoker for≤15 years | 4 |
| TCGA-HD-7831 | 0 | 0 | MALE | 74 | Stage III | Tongue | NA | Lifelong Non-smoker | NA |
| TCGA-HD-7832 | 0 | 0 | MALE | 52 | Stage IVA | Floor of Mouth | NA | Current smoker | 36 |
| TCGA-HD-7917 | 0 | 37 | MALE | 62 | Stage II | Floor of Mouth | NA | Current smoker | NA |
| TCGA-HD-8224 | 1 | 446 | MALE | 63 | Stage III | Base of Tongue | Negative | Lifelong Non-smoker | NA |
| TCGA-HD-8314 | 0 | 670 | MALE | 58 | Stage III | Base of Tongue | Positive | Lifelong Non-smoker | NA |
| TCGA-HD-8634 | 1 | 385 | FEMALE | 51 | Stage I | Tongue | Not Evaluated | Current smoker | 60 |
| TCGA-HD-8635 | 0 | 695 | FEMALE | 61 | Stage III | Tongue | Negative | Current smoker | 36 |
| TCGA-HD-A4C1 | 0 | 11 | FEMALE | 41 | Stage IVA | Buccal mucosa | Negative | Lifelong Non-smoker | NA |
| TCGA-HD-A633 | 0 | 421 | MALE | 74 | Stage IVA | Oral Cavity | Unknown | Lifelong Non-smoker | NA |
| TCGA-HD-A634 | 1 | 130 | MALE | 56 | Stage III | Tonsil | Positive | Current smoker | 40 |
| TCGA-HD-A6HZ | 0 | 111 | FEMALE | 79 | Stage II | Tongue | Unknown | Lifelong Non-smoker | NA |
| TCGA-HD-A6I0 | 0 | 210 | MALE | 56 | Stage III | Oral Cavity | Unknown | Current reformed smoker for> 15 years | 15 |
| TCGA-HL-7533 | 0 | 1057 | MALE | 65 | Stage IVA | Oral Cavity | NA | Current reformed smoker for> 15 years | 25 |
| TCGA-IQ-7630 | 0 | 485 | MALE | 49 | Stage III | Oropharynx | NA | Lifelong Non-smoker | NA |
| TCGA-IQ-7631 | 0 | 388 | FEMALE | 60 | Stage II | Buccal mucosa | NA | Current reformed smoker for> 15 years | 30 |
| TCGA-IQ-7632 | 0 | 441 | FEMALE | 68 | Stage IVA | Alveolar Ridge | NA | Current reformed smoker for> 15 years | 10 |
| TCGA-IQ-A61E | 0 | 588 | FEMALE | 55 | Stage III | Tongue | Not Evaluated | Current smoker | 45 |
| TCGA-IQ-A61G | 0 | 360 | MALE | 57 | Stage IVA | Floor of Mouth | Not Evaluated | Current reformed smoker for≤15 years | 60 |
| TCGA-IQ-A61H | 0 | 412 | MALE | 76 | Stage II | Tongue | Not Evaluated | Current reformed smoker for≤15 years | 30 |
| TCGA-IQ-A61I | 1 | 2 | MALE | 63 | Stage IVA | Oropharynx | Not Evaluated | Lifelong Non-smoker | NA |
| TCGA-IQ-A61J | 0 | 440 | MALE | 54 | Stage IVA | Tongue | Not Evaluated | Lifelong Non-smoker | NA |
| TCGA-IQ-A61O | 0 | 360 | MALE | 43 | Stage IVA | Oropharynx | Not Evaluated | Lifelong Non-smoker | NA |
| TCGA-KU-A66S | 1 | 406 | FEMALE | 69 | Stage III | Larynx | Not Evaluated | Current smoker | 50 |
| TCGA-KU-A66T | 0 | 552 | FEMALE | 53 | Stage IVA | Floor of Mouth | Not Evaluated | Lifelong Non-smoker | NA |
| TCGA-KU-A6H7 | 0 | 378 | FEMALE | 55 | Stage IVA | Tonsil | Positive | Current reformed smoker for≤15 years | 60 |
| TCGA-KU-A6H8 | 1 | 327 | MALE | 41 | Stage I | Tongue | Not Evaluated | Current smoker | 12.5 |
| TCGA-MT-A51W | 0 | 437 | FEMALE | 52 | Stage I | Tonsil | Negative | Current smoker | 10 |
| TCGA-MT-A51X | 0 | 242 | MALE | 30 | Stage IVA | Tongue | NA | Current smoker | 28.5 |
| TCGA-MT-A67A | 0 | 914 | FEMALE | 85 | Stage I | Tongue | Not Evaluated | Lifelong Non-smoker | NA |
| TCGA-MT-A67D | 0 | 56 | MALE | 55 | Stage II | Floor of Mouth | Not Evaluated | Current smoker | 40 |
| TCGA-MT-A67F | 0 | 384 | FEMALE | 60 | Stage IVA | Oral Cavity | Not Evaluated | Lifelong Non-smoker | NA |
| TCGA-MT-A7BN | 0 | 51 | MALE | 74 | Stage IVA | Floor of Mouth | Unknown | Lifelong Non-smoker | NA |
| TCGA-MZ-A5BI | 1 | 217 | MALE | 53 | Stage IVA | Tonsil | Positive | [Unknown] | NA |
| TCGA-MZ-A6I9 | 1 | 489 | MALE | 68 | Stage IVA | Base of Tongue | Positive | Current reformed smoker for> 15 years | 14 |
| TCGA-MZ-A7D7 | 0 | 240 | MALE | 51 | Stage IVA | Tonsil | Negative | Current smoker | 39 |
| TCGA-P3-A5Q5 | 0 | 910 | MALE | 54 | Stage IVA | Tonsil | Not Evaluated | Current reformed smoker for≤15 years | 30 |
| TCGA-P3-A5Q6 | 1 | 480 | MALE | 49 | Stage III | Tonsil | Not Evaluated | Current reformed smoker for≤15 years | 9 |
| TCGA-P3-A5QA | 0 | 1726 | MALE | 41 | Stage II | Tongue | Not Evaluated | Lifelong Non-smoker | NA |
| TCGA-P3-A5QE | 0 | 1559 | MALE | 49 | Stage III | Base of Tongue | Not Evaluated | Lifelong Non-smoker | NA |
| TCGA-P3-A5QF | 1 | 330 | MALE | 49 | Stage IVA | Alveolar Ridge | Not Evaluated | Lifelong Non-smoker | NA |
| TCGA-P3-A6SW | 0 | 1120 | MALE | 50 | Stage IVA | Tonsil | Not Evaluated | Current smoker | 20 |
| TCGA-P3-A6SX | 1 | 1430 | MALE | 67 | Stage IVA | Tonsil | Not Evaluated | Current reformed smoker for≤15 years | 100 |
| TCGA-P3-A6T0 | 0 | 578 | FEMALE | 47 | Stage IVA | Floor of Mouth | Not Evaluated | Current reformed smoker for≤15 years | 30 |
| TCGA-P3-A6T2 | 0 | 2298 | MALE | 45 | Stage II | Buccal mucosa | Not Evaluated | Lifelong Non-smoker | NA |
| TCGA-P3-A6T3 | 1 | 577 | MALE | 49 | Stage IVA | Oral Cavity | Not Evaluated | Lifelong Non-smoker | NA |
| TCGA-P3-A6T4 | 1 | 62 | MALE | 54 | Stage IVA | Floor of Mouth | Not Evaluated | Current smoker | 40 |
| TCGA-P3-A6T5 | 1 | 882 | FEMALE | 79 | Stage IVA | Alveolar Ridge | Not Evaluated | Current reformed smoker for> 15 years | 15 |
| TCGA-P3-A6T6 | 1 | 395 | MALE | 53 | Stage IVA | Alveolar Ridge | Not Evaluated | Lifelong Non-smoker | NA |
| TCGA-P3-A6T7 | 1 | 487 | MALE | 55 | Stage III | Floor of Mouth | Not Evaluated | Current smoker | 80 |
| TCGA-P3-A6T8 | 0 | 400 | MALE | 54 | Stage IVA | Floor of Mouth | Not Evaluated | Current smoker | 30 |
| TCGA-QK-A64Z | 1 | 641 | FEMALE | 79 | Stage II | Hard Palate | Not Evaluated | Lifelong Non-smoker | NA |
| TCGA-QK-A652 | 0 | 357 | MALE | 60 | Stage II | Tongue | Not Evaluated | Lifelong Non-smoker | NA |
| TCGA-QK-A6IF | 0 | 537 | MALE | 61 | Stage IVA | Tonsil | Positive | Lifelong Non-smoker | NA |
| TCGA-QK-A6IG | 1 | 222 | MALE | 69 | Stage II | Buccal mucosa | Not Evaluated | Current reformed smoker for≤15 years | 40 |
| TCGA-QK-A6IH | 0 | 508 | FEMALE | 65 | Stage IVA | Alveolar Ridge | Not Evaluated | Lifelong Non-smoker | NA |
| TCGA-QK-A6II | 1 | 284 | MALE | 52 | Stage III | Floor of Mouth | Not Evaluated | Current smoker | 30 |
| TCGA-QK-A6IJ | 0 | 387 | MALE | 71 | Stage II | Floor of Mouth | Not Evaluated | Current reformed smoker for≤15 years | 40 |
| TCGA-QK-A6V9 | 0 | 567 | MALE | 56 | Stage II | Tonsil | Positive | Lifelong Non-smoker | NA |
| TCGA-QK-A8Z7 | 0 | 198 | MALE | 59 | Stage IVA | Floor of Mouth | Not Evaluated | Current smoker | 8 |
| TCGA-QK-A8Z8 | 1 | 171 | FEMALE | 60 | Stage IVC | Larynx | Not Evaluated | Current smoker | 80 |
| TCGA-QK-A8Z9 | 0 | 352 | MALE | 56 | Stage IVA | Floor of Mouth | Not Evaluated | Current smoker | 35 |
| TCGA-QK-A8ZA | 0 | 368 | MALE | 60 | Stage IVA | Oropharynx | Positive | Current smoker | 15 |
| TCGA-QK-A8ZB | 0 | 296 | MALE | 68 | Stage IVA | Larynx | Not Evaluated | Current reformed smoker for> 15 years | NA |
| TCGA-QK-AA3J | 0 | 297 | MALE | 69 | Stage I | Larynx | Not Evaluated | Current reformed smoker for≤15 years | NA |
| TCGA-QK-AA3K | 0 | 253 | MALE | 60 | Stage IVA | Tongue | Positive | Current smoker | NA |
| TCGA-RS-A6TO | 1 | 387 | FEMALE | 82 | Stage IVA | Oral Cavity | Negative | Lifelong Non-smoker | NA |
| TCGA-RS-A6TP | 0 | 516 | MALE | 58 | Stage II | Tonsil | Positive | Current reformed smoker for≤15 years | 10 |
| TCGA-T2-A6WX | 1 | 209 | FEMALE | 73 | Stage III | Floor of Mouth | Not Evaluated | Lifelong Non-smoker | NA |
| TCGA-T2-A6WZ | 1 | 484 | MALE | 53 | Stage IVA | Base of Tongue | Not Evaluated | Current smoker | 40 |
| TCGA-T2-A6X0 | 0 | 216 | MALE | 49 | Stage IVA | Tonsil | Not Evaluated | Current reformed smoker for≤15 years | 10 |
| TCGA-TN-A7HI | 0 | 412 | MALE | 56 | Stage I | Tonsil | Positive | Current smoker | 16 |
| TCGA-TN-A7HJ | 0 | 403 | MALE | 51 | Stage III | Larynx | Not Evaluated | Lifelong Non-smoker | NA |
| TCGA-TN-A7HL | 0 | 416 | MALE | 59 | Stage IVA | Hypopharynx | Positive | Current reformed smoker for≤15 years | 10 |
| TCGA-UF-A718 | 0 | 1326 | MALE | 62 | Stage IVA | Larynx | Not Evaluated | Current smoker | 50 |
| TCGA-UF-A719 | 0 | 1663 | MALE | 54 | Stage III | Floor of Mouth | Not Evaluated | Current smoker | NA |
| TCGA-UF-A71A | 1 | 86 | MALE | 67 | Stage IVA | Floor of Mouth | Not Evaluated | Current reformed smoker for> 15 years | NA |
| TCGA-UF-A71B | 0 | 1319 | MALE | 50 | Stage IVA | Alveolar Ridge | Not Evaluated | Current smoker | 30 |
| TCGA-UF-A71D | 0 | 1252 | FEMALE | 54 | Stage IVA | Larynx | Not Evaluated | Current smoker | 22 |
| TCGA-UF-A71E | 0 | 1278 | MALE | 63 | Stage IVA | Floor of Mouth | Not Evaluated | Lifelong Non-smoker | NA |
| TCGA-UF-A7J9 | 0 | 1043 | MALE | 75 | Stage IVA | Larynx | Not Evaluated | Current smoker | 25.5 |
| TCGA-UF-A7JA | 0 | 1894 | FEMALE | 66 | Stage IVA | Buccal mucosa | Not Evaluated | Current smoker | NA |
| TCGA-UF-A7JC | 1 | 546 | MALE | 42 | Stage III | Floor of Mouth | Not Evaluated | Current smoker | 38 |
| TCGA-UF-A7JD | 0 | 735 | MALE | 71 | Stage IVA | Buccal mucosa | Not Evaluated | Current smoker | 61 |
| TCGA-UF-A7JF | 0 | 1497 | MALE | 80 | Stage IVA | Larynx | Not Evaluated | Current smoker | 42 |
| TCGA-UF-A7JH | 0 | 665 | MALE | 59 | Stage IVA | Larynx | Not Evaluated | Current reformed smoker for≤15 years | 52 |
| TCGA-UF-A7JJ | 0 | 490 | MALE | 68 | Stage IVA | Larynx | Not Evaluated | Current smoker | 80 |
| TCGA-UF-A7JK | 1 | 424 | MALE | 59 | Stage IVA | Larynx | Not Evaluated | Current reformed smoker for≤15 years | NA |
| TCGA-UF-A7JS | 1 | 680 | MALE | 59 | Stage IVA | Tongue | Not Evaluated | Current smoker | 25.5 |
| TCGA-UF-A7JV | 1 | 90 | FEMALE | 62 | Stage IVA | Hypopharynx | Not Evaluated | Lifelong Non-smoker | NA |
| TCGA-WA-A7GZ | 1 | 625 | MALE | 58 | Stage II | Floor of Mouth | NA | Current smoker | 20 |
| TCGA-WA-A7H4 | 0 | 443 | MALE | 69 | Stage II | Tongue | NA | Current smoker | 25 |

**Table S2** Univariate Cox regression analysis with a significance level of 0.001 reveals a significant relation between mRNA expression and survival time, and the *t* test with differential expression between patients with longer OS and those with shorter OS time.

| **Gene_id** | **P-value^a^** | **logFC** | **AveExpr** | **P.Value^b^** |
| --- | --- | --- | --- | --- |
| TMED7-TICAM2\|100302736 | 3.20E-05 | 1.113 | 7.177 | 7.68E-05 |
| ERRFI1\|54206 | 5.10E-04 | 0.948 | 10.655 | 1.29E-04 |
| CDC27\|996 | 4.50E-06 | 0.364 | 10.721 | 2.76E-04 |
| TREX1\|11277 | 1.00E-03 | -0.579 | 8.321 | 5.49E-04 |
| AREG\|374 | 2.50E-04 | 1.88 | 9.256 | 6.10E-04 |
| FGD3\|89846 | 4.70E-07 | -0.969 | 7.423 | 6.50E-04 |
| SLC20A1\|6574 | 2.00E-04 | 0.587 | 10.545 | 8.09E-04 |
| CCBE1\|147372 | 2.40E-04 | 2.074 | 4.572 | 9.36E-04 |
| PDGFA\|5154 | 1.80E-04 | 0.916 | 9.31 | 1.04E-03 |
| C17orf44\|284029 | 7.40E-04 | -0.78 | 5.173 | 1.16E-03 |
| TCP11\|6954 | 3.50E-04 | -1.915 | 2.006 | 1.52E-03 |
| TRAK1\|22906 | 6.60E-04 | -0.403 | 10.288 | 2.28E-03 |
| PLAU\|5328 | 4.60E-04 | 1.003 | 12.555 | 2.50E-03 |
| CTTN\|2017 | 9.20E-04 | 1.034 | 13.155 | 2.51E-03 |
| PRRT3\|285368 | 4.00E-04 | -0.63 | 5.182 | 2.62E-03 |
| SLC25A45\|283130 | 1.40E-05 | -0.625 | 5.771 | 2.98E-03 |
| PTX3\|5806 | 3.50E-04 | 1.871 | 4.773 | 3.03E-03 |
| D2HGDH\|728294 | 1.60E-04 | -0.689 | 7.594 | 3.70E-03 |
| MT2A\|4502 | 6.40E-04 | 1.402 | 12.509 | 3.77E-03 |
| SNX14\|57231 | 7.40E-05 | 0.436 | 9.551 | 4.42E-03 |
| NT5E\|4907 | 9.60E-04 | 1.501 | 9.865 | 4.62E-03 |
| FADD\|8772 | 3.60E-04 | 1.09 | 10.359 | 4.62E-03 |
| IP6K2\|51447 | 9.80E-04 | -0.38 | 9.714 | 4.75E-03 |
| TSPAN32\|10077 | 2.70E-04 | -0.913 | 2.534 | 5.10E-03 |
| UNK\|85451 | 7.20E-04 | -0.328 | 9.173 | 6.05E-03 |
| TMC8\|147138 | 5.80E-04 | -0.822 | 8.175 | 6.36E-03 |
| ITGA5\|3678 | 2.60E-04 | 0.873 | 12.314 | 6.47E-03 |
| CSF2\|1437 | 4.10E-04 | 1.66 | 4.666 | 6.59E-03 |
| BCAR3\|8412 | 6.20E-04 | 0.977 | 9.267 | 6.65E-03 |
| MAGIX\|79917 | 3.60E-04 | -0.675 | 4.875 | 7.35E-03 |
| C19orf57\|79173 | 7.80E-05 | -0.797 | 5.552 | 7.44E-03 |
| uc001vqg.2 | 4.80E-04 | 1.501 | 4.065 | 7.56E-03 |
| LOC728743\|728743 | 4.60E-04 | -0.642 | 5.889 | 7.73E-03 |
| SEMA3A\|10371 | 3.20E-05 | 1.319 | 7.359 | 7.78E-03 |
| MPND\|84954 | 1.70E-04 | -0.482 | 8.195 | 8.79E-03 |
| uc004dek.1 | 4.20E-04 | -0.749 | 1.28 | 9.02E-03 |
| ZSWIM4\|65249 | 1.00E-03 | -0.505 | 8.985 | 9.48E-03 |
| MEIS1\|4211 | 2.90E-04 | -0.787 | 7.493 | 9.52E-03 |
| PCMT1\|5110 | 6.90E-05 | 0.39 | 10.53 | 9.92E-03 |
| ZNF266\|10781 | 1.90E-05 | -0.46 | 8.449 | 1.05E-02 |
| MORF4L2\|9643 | 1.40E-04 | 0.323 | 12.189 | 1.06E-02 |
| ZNF541\|84215 | 9.00E-05 | -1.559 | 2.178 | 1.07E-02 |
| EFNB2\|1948 | 1.10E-05 | 0.595 | 10.9 | 1.16E-02 |
| RAF1\|5894 | 1.30E-04 | -0.235 | 10.662 | 1.28E-02 |
| uc003wbm.3 | 9.90E-04 | -1.467 | 4.413 | 1.30E-02 |
| MAP7D3\|79649 | 1.30E-04 | 0.425 | 8.435 | 1.35E-02 |
| FST\|10468 | 9.00E-04 | 1.038 | 10.273 | 1.40E-02 |
| KIAA1875\|340390 | 3.90E-04 | -1.112 | 2.412 | 1.59E-02 |
| CAMK2N1\|55450 | 2.20E-04 | 0.931 | 8.667 | 1.66E-02 |
| KIAA1683\|80726 | 7.70E-05 | -1.191 | 3.849 | 1.67E-02 |
| SSR3\|6747 | 3.60E-04 | 0.342 | 11.87 | 1.74E-02 |
| SEMA3C\|10512 | 3.10E-04 | 0.852 | 11.006 | 1.76E-02 |
| FRAT1\|10023 | 1.20E-04 | -0.554 | 5.905 | 1.80E-02 |
| YIPF4\|84272 | 4.60E-04 | 0.65 | 9.5 | 1.91E-02 |
| ZAP70\|7535 | 2.50E-04 | -1.024 | 5.742 | 1.92E-02 |
| AMIGO3\|386724 | 3.60E-04 | -0.519 | 5.798 | 1.93E-02 |
| ZNF324B\|388569 | 6.60E-04 | -0.809 | 6.96 | 2.08E-02 |
| CAV2\|858 | 1.20E-04 | 0.564 | 11.653 | 2.11E-02 |
| C19orf6\|91304 | 7.90E-04 | -0.305 | 11.108 | 2.21E-02 |
| S1PR4\|8698 | 2.40E-04 | -0.713 | 5.529 | 2.22E-02 |
| uc010tmr.1 | 7.50E-04 | -0.759 | 1.344 | 2.34E-02 |
| PPP1R3E\|90673 | 7.40E-04 | -0.584 | 4.739 | 2.40E-02 |
| WDR48\|57599 | 3.10E-04 | -0.339 | 9.161 | 2.49E-02 |
| PSMA1\|5682 | 1.10E-04 | 0.301 | 11.325 | 2.59E-02 |
| TTC21A\|199223 | 8.20E-05 | -0.495 | 4.021 | 2.62E-02 |
| TMEM87A\|25963 | 7.40E-04 | 0.273 | 11.037 | 2.76E-02 |
| FRMD5\|84978 | 3.10E-04 | 1.21 | 3.716 | 2.78E-02 |
| ARRDC5\|645432 | 2.70E-04 | -0.519 | 1.148 | 2.81E-02 |
| VILL\|50853 | 7.90E-04 | -0.796 | 6.547 | 2.87E-02 |
| C15orf53\|400359 | 5.90E-04 | -0.363 | 0.619 | 2.94E-02 |
| PDIA3P\|171423 | 1.30E-05 | 0.264 | 12.266 | 3.02E-02 |
| YWHAG\|7532 | 8.40E-04 | 0.317 | 13.013 | 3.26E-02 |
| NISCH\|11188 | 7.30E-04 | -0.318 | 9.834 | 3.35E-02 |
| SAR1A\|56681 | 7.10E-04 | 0.248 | 11.258 | 3.55E-02 |
| BRPF1\|7862 | 2.20E-04 | -0.282 | 8.961 | 3.77E-02 |
| C3orf70\|285382 | 5.50E-04 | -0.887 | 5.543 | 3.77E-02 |
| TPCN2\|219931 | 1.00E-03 | 0.647 | 8.717 | 3.82E-02 |
| PDIA3\|2923 | 5.90E-05 | 0.258 | 13.089 | 3.87E-02 |
| CIAPIN1\|57019 | 4.30E-04 | 0.236 | 10.152 | 3.94E-02 |
| ATP9B\|374868 | 4.70E-04 | -0.401 | 8.231 | 3.98E-02 |
| RAB11B\|9230 | 8.10E-05 | -0.263 | 10.805 | 4.05E-02 |
| PSMD7\|5713 | 2.20E-05 | 0.22 | 11.53 | 4.29E-02 |
| ADA\|100 | 3.80E-04 | 0.528 | 9.088 | 4.43E-02 |
| C3orf19\|51244 | 4.70E-04 | -0.217 | 8.008 | 4.52E-02 |
| RABEP2\|79874 | 7.10E-04 | -0.321 | 8.765 | 4.53E-02 |
| CD27\|939 | 7.70E-04 | -0.984 | 6.429 | 4.68E-02 |
| MRPL21\|219927 | 5.10E-04 | 0.518 | 9.591 | 4.77E-02 |
| uc001dqd.1 | 7.20E-04 | 1.029 | 2.88 | 4.81E-02 |
| CELSR3\|1951 | 1.80E-04 | -0.905 | 7.078 | 4.82E-02 |
| PRDM15\|63977 | 1.00E-03 | -0.311 | 7.783 | 4.90E-02 |
| PARP15\|165631 | 6.10E-04 | -0.812 | 3.071 | 4.98E-02 |
| **a** log-rank test in Univariate Cox regression analysis  **b** t-test | | | | |
